# Late-life targeting of the IGF-1 receptor improves healthspan and lifespan in female mice

**DOI:** 10.1038/s41467-018-04805-5

**Published:** 2018-06-19

**Authors:** Kai Mao, Gabriela Farias Quipildor, Tahmineh Tabrizian, Ardijana Novaj, Fangxia Guan, Ryan O. Walters, Fabien Delahaye, Gene B. Hubbard, Yuji Ikeno, Keisuke Ejima, Peng Li, David B. Allison, Hossein Salimi-Moosavi, Pedro J. Beltran, Pinchas Cohen, Nir Barzilai, Derek M. Huffman

**Affiliations:** 1 Department of Molecular Pharmacology, Albert Einstein College of Medicine, Bronx, NY 10461 USA; 2 Department of Medicine, Albert Einstein College of Medicine, Bronx, NY 10461 USA; 3 Institute for Aging Research, Albert Einstein College of Medicine, Bronx, NY 10461 USA; 4 Department of Genetics, Albert Einstein College of Medicine, Bronx, NY 10461 USA; 5 Obstetrics and Gynecology and Women’s Health, Albert Einstein College of Medicine, Bronx, 10461 NY USA; 6 Barshop Institute for Longevity and Aging Studies and Department of Pathology, University of Texas Health Science Center at San Antonio, San Antonio, TX 78245 USA; 7 Geriatric Research, Education & Clinical Center (GRECC), Audie L. Murphy Memorial VA Hospital, San Antonio, TX 78229 USA; 8 School of Health Professions, The University of Alabama at Birmingham, Birmingham, AL 35294 USA; 9 School of Public Health, Indiana University, Bloomington, IN 47405 USA; 10 School of Public Health, The University of Alabama at Birmingham, Birmingham, AL 35294 USA; 11 Department of Pharmacokinetics and Drug Metabolism, Amgen Inc., Thousand Oaks, CA 91320 USA; 12 Oncology Research, Amgen Inc., Thousand Oaks, CA 91320 USA; 13 Davis School of Gerontology, University of Southern California, Los Angeles, CA 90089 USA

## Abstract

Diminished growth factor signaling improves longevity in laboratory models, while a reduction in the somatotropic axis is favorably linked to human aging and longevity. Given the conserved role of this pathway on lifespan, therapeutic strategies, such as insulin-like growth factor-1 receptor (IGF-1R) monoclonal antibodies (mAb), represent a promising translational tool to target human aging. To this end, we performed a preclinical study in 18-mo-old male and female mice treated with vehicle or an IGF-1R mAb (L2-Cmu, Amgen Inc), and determined effects on aging outcomes. Here we show that L2-Cmu preferentially improves female healthspan and increases median lifespan by 9% (*P* = 0.03) in females, along with a reduction in neoplasms and inflammation (*P* ≤ 0.05). Thus, consistent with other models, targeting IGF-1R signaling appears to be most beneficial to females. Importantly, these effects could be achieved at advanced ages, suggesting that IGF-1R mAbs could represent a promising therapeutic candidate to delay aging.

## Abstract (web summary)

Reduced IGF-1 signaling increases longevity in many organisms. Here, Mao et al. show that administration of an anti-IGF-1R antibody is well tolerated and delays aging in female mice; importantly, late-life targeting is sufficient to achieve the beneficial effects.

## Introduction

Diminished growth hormone (GH) and insulin/insulin-like growth factor-1 (IGF-1) signaling extends lifespan in many laboratory models, including mutations to *daf2* in worms^1^, *Sch9* in yeast^2^, and *Chico* in *drosophila*^3^. Likewise, several dwarf models, including Ames, Snell and growth hormone receptor knockout (GHRKO) mice, are exceptionally long lived^4,5^. A specific role for IGF-1 receptor (IGF-1R) signaling in the mediation of mammalian longevity was first established in *IGF-1R* haploinsufficient mice, which lived 33% longer than controls, but unlike other models of reduced somatotropic signaling, this effect was female specific^6^. This unique sex difference was subsequently confirmed in two follow-up studies, though with more modest reported improvements in female lifespan^7,8^, while a life shortening effect was observed in males^8^. The underlying mechanism(s) linking reduced IGF-1 signaling to improved mammalian lifespan is thought to involve improved stress defenses and lower risk for proliferative diseases^9–11^, though the reason for sex differences in this response remains unresolved.

Several examples have also now emerged suggesting the GH/IGF-1 signaling pathway is relevant to human aging^12^, including the discovery of functional mutations in the *IGF-1R* gene in individuals with exceptional longevity, resulting in relative IGF-1 resistance^13,14^, and in subjects lacking functional GH receptors (Laron dwarfs)^15^. Remarkably, low IGF-1 levels also predict better survival in nonagenarians, and similar to lessons learned in *IGF-1R* heterozygous mice, this effect is female specific^16^. Likewise, higher circulating levels of IGF-1 have been consistently associated with multiple site-specific cancers in epidemiologic studies^12^. Thus, given the accumulating evidence across species implicating this pathway as integral to aging and its associated diseases, the development of therapeutics aimed at modulating IGF-1 signaling in humans could prove highly effective as a translational tool to delay aging. However, given that previous demonstrations of longevity resulting from disruption of this pathway occurred either at conception or in young adulthood^6,7,17–19^, along with the reported importance of low exposure to GH (and IGF-1) signals early in life on longevity and related outcomes^20,21^, whether benefits can be achieved by targeting this pathway later in life is unclear.

Anti-IGF-1 receptor (IGF-1R) monoclonal antibodies (mAbs) were developed for clinical use in treating advanced stage cancers^22–24^, including Ganitumab, which remains under investigation as a combination therapy in clinical trials targeting Rhabdomyosarcoma (NCT03041701) and Ewing Sarcoma (NCT02306161), for which it recently received orphan drug status. We therefore postulated that IGF-1R mAbs could represent a viable therapeutic tool to target IGF-1 action, and potentially mimic the beneficial effects associated with diminished IGF-1 signaling observed in animal models. In order to test this possibility, we engineered a murinized version of the anti-IGF-1R mAb, L2-C (L2-Cmu), in order to reduce effector function and enable chronic administration in mice. Here, we provide the first evidence of delayed aging with a therapeutic mAb, via long-term modulation of IGF-1 action. L2-Cmu proved feasible and well tolerated in older animals, and consistent with genetic models of *IGF-1R* heterozygosity^6–8^, improves female healthspan and lifespan. Importantly, these effects were achieved even though treatment was not initiated until 18 mo of age. Thus, these data suggest that late-life targeting of IGF-1R signaling can recapitulate effects observed in genetic models of constitutive *IGF-1R* haploinsufficiency on lifespan. As IGF-1R mAbs are readily available for human use, these observations warrant further study into potentially harnessing these drugs to target at least some manifestations of aging.

## Results

### L2-Cmu is a selective antagonist to the IGF-1R and hybrids

**Fig. 1:**
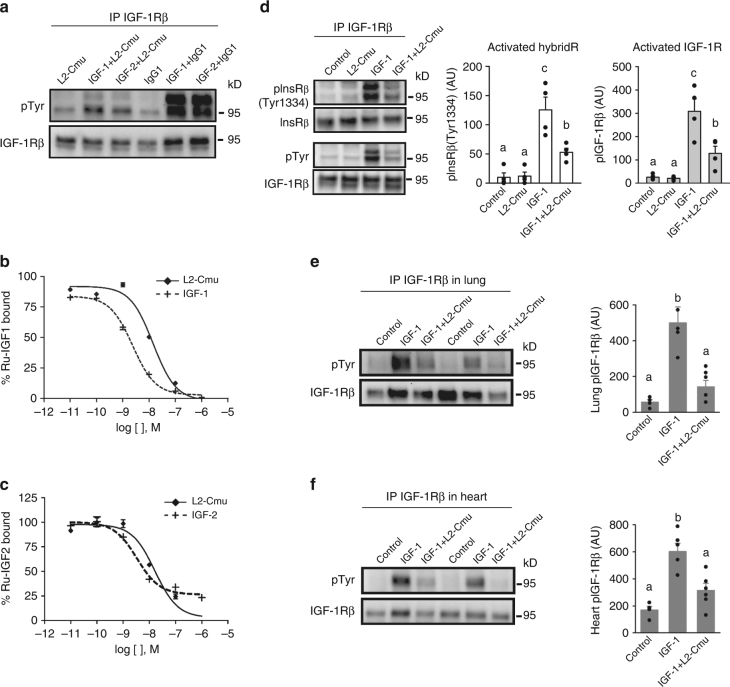
L2-Cmu is an antagonist to the murine IGF-1R and hybrids. **a** NIH-3T3 cells were pre-treated with 100 μg/mL Control IgG1 or L2-Cmu for 1 h prior to addition of vehicle, 5 nM IGF-1 or 20 nM IGF-2 for 2 min. Cell lysates were then immunoprecipitated for IGF-1Rβ and blots were probed for pTyr and total IGF-1Rβ levels. **b**, **c** Dose response competition assays with murine IGF-1R(ECD)-mFc. The ability of L2-Cmu to block human Ru-labeled IGF-1 or IGF-2 to the murine IGF-1R extracellular domain was evaluated in the IGEN format. **d** NIH-3T3 cells were pre-treated with vehicle or L2-Cmu (100 µg/mL) 1 h prior to addition of media (Con), or IGF-1 (5 nM) for 2 min to assess activation of IGF-1R and IGF-1R/InsR Hybrids. Data show that L2-Cmu reduced activation of IGF-1Rs, HybridRs by ~65% as compared to IGF-1 alone (*n* = 4 per condition). **e**, **f** For in vivo validation, mice were pre-treated with vehicle or L2-Cmu (20 mg/kg) i.p. and then challenged with an i.v. bolus of saline or 5 µg IGF-1 to assess the ability of L2-Cmu to modulate IGF-1R activation in vivo for a total of three experimental groups: Control (vehicle + saline; *n* = 4), IGF-1 (vehicle + IGF-1; *n* = 5), and IGF-1 plus L2-Cmu (*n* = 5 for lung and *n* = 6 for heart). L2-Cmu significantly inhibited IGF-1R activation in lung and heart. Bars represent mean ± s.e.m. Dot plots overlaid on bar graphs represent individual data points. Different letters denote a significant difference between treatments by Tukey HSD, *P* ≤ 0.05

L2-Cmu was developed as a murinized version of the L2-C mAb at Amgen Inc. (Thousand Oaks, CA)^25^. Western blotting and Biacore analysis confirmed that L2-Cmu binds to and inhibits IGF-1R activation by IGF-1 (Ki = 3.3 nM) and IGF-2 (Ki = 3.3 nM) (Fig. 1a; Supplementary Table 1), which was verified in the IGEN format (Fig. 1b, c). In NIH-3T3 mouse fibroblasts cells, pre-treatment with L2-Cmu led to an ~65% inhibition of IGF-1-mediated activation of IGF-1Rs and InsR/IGF-1R hybrid receptors (HybridRs) (Fig. 1d; *P* < 0.05). Moreover, when mice were pre-treated with vehicle or L2-Cmu (20 mg/kg) by intraperitoneal (i.p.) injection, and then challenged with an intravenous (i.v.) bolus of saline or 5 µg IGF-1, L2-Cmu was able to modulate IGF-1R activation in vivo, as demonstrated by a near complete inhibition of IGF-1R phosphorylation in lung and ~70% inhibition in heart (Fig. 1e, f; *P* < 0.05).

### Chronic L2-Cmu treatment is well tolerated in older mice

We next performed a 6 mo feasibility study with weekly L2-Cmu i.p. injections (20 mg/kg) in 18-mo-old CB6F1 male and female mice, to carefully characterize the safety and efficacy of chronic IGF-1R modulation in aging animals. This dose of L2-Cmu was based in part on a prior pharmacodynamic (PD) study in which L2-C achieved similar suppression of tumor growth in vivo as Ganitumab^25^. A pharmacokinetic (PK) study confirmed L2-Cmu levels after a single injection were ~5-fold greater than the reported IC_90_ values for Ganitumab (28 µg/mL) in both sexes for at least 7 days (Supplementary Fig. 1a), while chronic exposure levels of L2-Cmu were similarly increased to levels expected to provide sufficient coverage in vivo (Supplementary Fig. 1b). In females, L2-Cmu mAb treatment led to a slight, albeit significant reduction in red blood cells (RBCs), hemoglobin (Hb), hematocrit (Hct), white blood cells (WBCs), lymphocytes (Supplementary Table 2), and serum phosphorus (Supplementary Table 3), though most values, with the exception of WBCs, remained within the “normal range”, and were less severely affected than previously reported with Ganitumab in young CD1 mice^26^. In males, no significant effects of L2-Cmu were observed on WBCs (Supplementary Table 4) or RBCs (Supplementary Table 4), but total protein, globulin, and ALT were slightly increased, while serum creatinine was reduced (Supplementary Table 5).

### L2-Cmu does not perturb glucose homeostasis in older mice

**Fig. 2:**
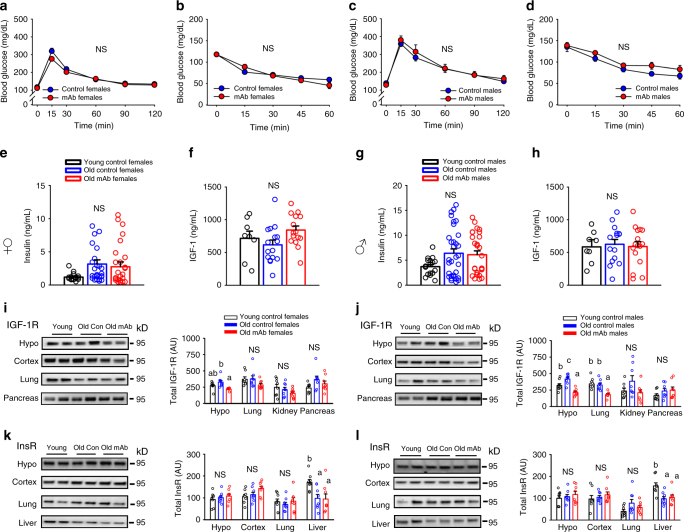
L2-Cmu does not perturb glucose homeostasis in aged mice. **a**–**d** Glucose and insulin tolerance in 24-mo-old female and male mice is not adversely effected by 6 mo mAb treatment (*n* = 12 per group, per sex). **e**–**h** Chronic L2-Cmu treatment led to a non-significant (main effect, *P* = 0.097), numerical increase in plasma IGF-1 level in old females [Young (*n* = 8), Old Con (*n* = 16), and Old mAb (*n* = 15)], while no significant difference was observed for IGF-1 levels in male mice [Young (*n* = 8), Old Con (*n* = 15), and Old mAb (*n* = 16)]. Likewise, insulin levels were not significantly different in female [Young (*n* = 16), Old Con (*n* = 26), and Old mAb (*n* = 25)], or male mice [Young (*n* = 17), Old Con (*n* = 32), and Old mAb (*n* = 28)]. **i**, **j** L2-Cmu treatment in males and females prevented the age-related increase in hypothalamic IGF-1Rs and reduced cortical levels in males only (*P* ≤ 0.05), but had no effect on IGF-1Rs in lung or pancreas of either sex. **k**, **l** Further, L2-Cmu treatment had no effect on InsR levels in examined tissues of either sex (*n* = 8 per group, per sex). Bars represent mean ± s.e.m. Dot plots overlaid on bar graphs represent individual data points. NS not significant. Different letters denote a significant difference between groups by Tukey HSD, *P* ≤ 0.05

We then evaluated effects of chronic IGF-1R mAb treatment on energy and glucose homeostasis in an initial cohort of older mice. In females, no significant effect on body weight, composition or energy balance was observed following 6 mo of L2-Cmu treatment (Supplementary Fig. 2a-f). In males, body weight was numerically decreased (*P* = 0.163) while lean mass was significantly reduced with mAb treatment (Supplementary Fig. 2g-h; *P* = 0.006), without effects on adiposity (Supplementary Fig. 2i), while energy expenditure was slightly reduced, without affecting food intake (Supplementary Fig. 2j-k; *P* ≤ 0.05). An increase in the respiratory exchange ratio (RER), indicative of increased carbohydrate utilization, was detected in mAb-treated males (Supplementary Fig. 2l; *P* ≤ 0.05). However, mAb treatment did not perturb glucose homeostasis (Fig. 2a–d) or insulin levels (Fig. 2e, g) in males or females. L2-Cmu led to a modest, numerical increase in circulating IGF-1 levels in older females (Fig. 2f; main effect *P* = 0.097), while IGF-1 levels in males were unaffected by treatment or age in this strain (Fig. 2h).

Interestingly, mAb treatment prevented the age-related rise in hypothalamic IGF-1R levels in both sexes, while resulting in reduced cortical IGF-1R levels in males only (Fig. 2i, j*; P* ≤ 0.05), without significant effects on IGF-1R levels in lung or pancreas (Fig. 2i, j). Meanwhile, InsR levels were unaffected by mAb treatment in all tissues examined for either sex (Fig. 2k, l). Likewise, no effect was observed on downstream components of the Insulin/IGF-1 signaling pathway with mAb treatment in females (Supplementary Fig 3), but L2-Cmu attenuated S6 activation in male hypothalamus (Supplementary Fig 4c; *P* ≤ 0.05).

### L2-Cmu preferentially benefits female healthspan

**Fig. 3:**
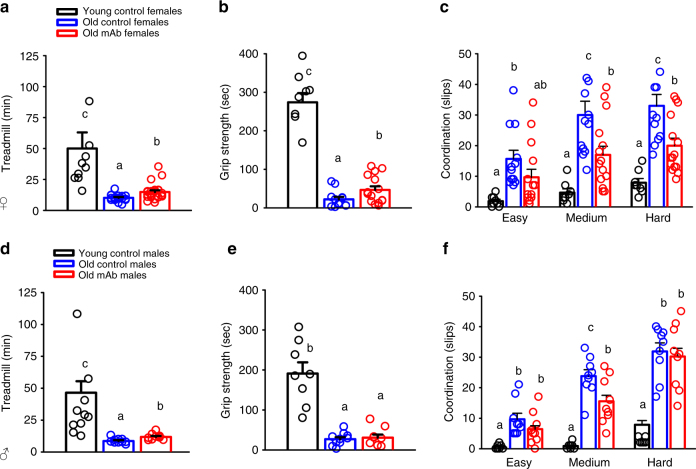
L2-Cmu preferentially improves female healthspan. Following 6 mo of mAb treatment, several functional measures of healthspan were assessed, including exercise tolerance in female [Young (*n* = 8), Old Con (*n* = 16), and Old mAb (*n* = 15)] and male mice [Young (*n* = 10), Old Con (*n* = 9), and Old mAb (*n* = 9)], grip strength in female [Young (*n* = 8), Old Con (*n* = 12), and Old mAb (*n* = 9)] and male mice [Young (*n* = 10), Old Con (*n* = 10), and Old mAb (*n* = 9)], and balance beam in female [Young (*n* = 8), Old Con (*n* = 12), and Old mAb (*n* = 15)] and male mice [Young (*n* = 8), Old Con (*n* = 9), and Old mAb (*n* = 9)]. **a**–**c** In females, mAb led to significant improvements in exercise tolerance (~50% over Old Con), grip strength (two-fold over Old Con), and motor coordination. **d**–**f** In males, L2-Cmu improved exercise tolerance by ~28%, but did not lead to improvements in grip strength, while motor coordination was only modestly improved on a medium difficulty beam. Bars represent mean ± s.e.m. Dot plots overlaid on bar graphs represent individual data points. Different letters denote a significant difference between groups by Tukey HSD, *P* ≤ 0.05

We next evaluated effects on functional healthspan domains related to neuromuscular and physical performance following 5–6 mo of mAb treatment. With aging, females (Fig. 3a–c) and males (Fig. 3d–f) demonstrated characteristic declines in endurance, strength, and motor coordination. However, as compared to Old Con females, age-matched L2-Cmu-treated females had ~50% greater exercise tolerance (Fig. 3a; *P* ≤ 0.05), a two-fold increase in grip strength (Fig. 3b; *P* ≤ 0.05), and improved gross motor coordination, by reducing the number of slips on a medium and hard difficulty balance beam (Fig. 3c; *P* ≤ 0.05). In males, declining exercise tolerance was modestly mitigated with mAb treatment (~28% greater than Old Con males; Fig. 3d; *P* ≤ 0.05), but no effect was observed on strength (Fig. 3e), and coordination was only marginally improved on a medium (Fig. 3f; *P* ≤ 0.05), but not hard difficulty beam (Fig. 3f).

### L2-Cmu treatment improves female cardiac function

**Fig. 4:**
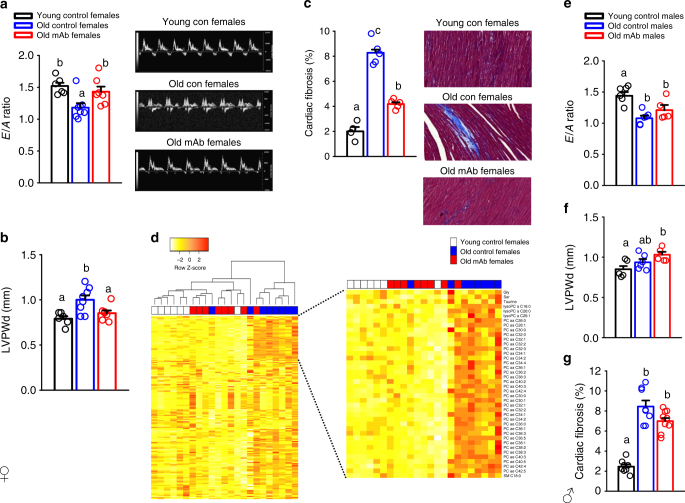
L2-Cmu prevents age-related diastolic dysfunction in females. **a**–**c** Cardiac aging in CB6F1 female mice is characterized by a decline in diastolic function (E/A ratio), increased LVPWd, and accumulating amounts of fibrosis in the myocardium. However, 6 mo L2-Cmu treatment, beginning at 78 wks of age, was able to preserve diastolic function, reduce LVPWd measures in females [Young (*n* = 6), Old Con (*n* = 8), and Old mAb (*n* = 7)], and largely prevented the accumulation of cardiac fibrosis [Young (*n* = 4), Old Con (*n* = 5), and Old mAb (*n* = 6)]. **d** Metabolomic analysis of heart tissue in female mice [Young (*n* = 7), Old Con (*n* = 8), and Old mAb (*n* = 7)] revealed that the young and aged metabolome are distinct (see also Supplementary Fig. 5), and predominantly characterized by differences in glycerophospholipids, and to a lesser extent ceramides, while amino acids, biogenic amines, and acylcarnitines were largely unaffected. However, mAb treatment tended to oppose the age-related alterations in several metabolites, including the rise of several glycerophospholipids, resulting in a more youthful metabolomic signature in heart. Further metabolomic data analyses can be found in in Supplementary Fig. 5 and the dataset here: [10.17605/OSF.IO/8QGX9]. **e**–**g** Remarkably, the favorable effects of L2-Cmu in males were absent for E/A ratio [Young (*n* = 5), Old Con (*n* = 6), and Old mAb (*n* = 5)] and fibrosis [Young (*n* = 8), Old Con (*n* = 8), and Old mAb (*n* = 8)], suggesting that the beneficial effects of modulating IGF-1 signaling in heart is specific to females. Bars represent mean ± s.e.m. Dot plots overlaid on bar graphs represent individual data points. Different letters denote a significant difference between groups by Tukey HSD, *P* ≤ 0.05

Given the reported importance of IGF-1 signaling to the myocardium^27,28^, we next assessed the effects of IGF-1R antagonism on cardiovascular function by echocardiography (Fig. 4). Cardiac aging in mice is characterized by a decline in diastolic function^29^, which we confirmed by a reduction in the E/A ratio with age in both sexes (Fig. 4a, e; *P* ≤ 0.05). Importantly, L2-Cmu treatment in females did not adversely affect cardiac function, but instead restored diastolic function to more youthful levels (Fig. 4a), and this was associated with a reduction in measures of left ventricular posterior wall end diastole (LVPWd; Fig. 4b; *P* ≤ 0.05) and cardiac fibrosis (Fig. 4c; *P* ≤ 0.05). However, unlike a recent report where constitutive loss of IGF-1Rs specifically in the myocardium of male mice prevented age-related alterations to the heart^30^, late-life mAb treatment failed to preserve or restore these same parameters in male animals (Fig. 4e-g).

Because rapamycin, which extends mouse lifespan^31^, can reverse age-related diastolic dysfunction and restore a more youthful metabolome in the aged myocardium^32^, we next evaluated metabolomic changes in Young, Old Con, and Old mAb female-treated hearts. We observed that more than one-third of metabolites detected in heart were significantly altered with aging, which was predominantly characterized by a rise in the level of glycerophospholids, and to a lesser extent, acylcarnitines (Fig. 4d; *P* ≤ 0.05). However, mAb treatment tended to oppose age-related changes in 30 of these metabolites (22 marginally significant after FDR correction), including a reduction in the age-related rise of many glycerophospholipids, and a distinct clustering of metabolites from heart tissue in Old Con versus Old mAb-treated animals by PCA (Supplementary Fig. 5), resulting in a more youthful metabolomic signature in heart.

### Sexually-dimorphic effects of L2-Cmu on inflammatory markers

**Fig. 5:**
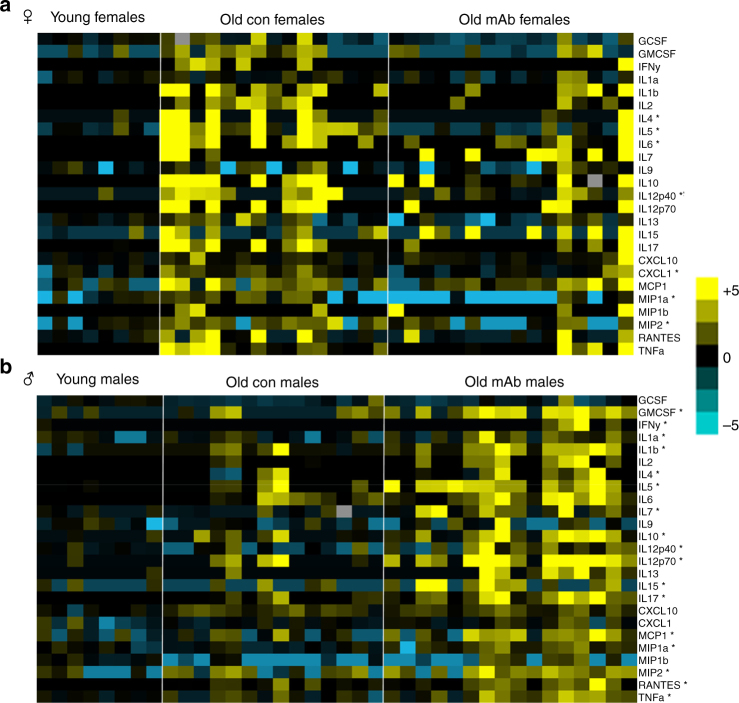
Sex differences in inflammatory markers with IGF-1R mAb treatment in aged mice. **a**, **b** A 25-plex cytokine/chemokine panel was performed on plasma obtained from female [Young (*n* = 8), Old Con (*n* = 15), and Old mAb (*n* = 16)] and male mice [Young (*n* = 8), Old Con (*n* = 14), and Old mAb (*n* = 16)]. Data were treated as nonparametric values and analyzed by the Kruskal–Wallis procedure and the Mann–Whitney *U*-test when appropriate. Any value below the lower limit of detection of the assay was replaced by the minimal detectable concentration (MOD)/√2 for the specific analyte. Therefore, undetectable values were treated as a tie for purposes of statistically ranking data. For generation of the heatmaps, values were normalized against Young Controls and log transformed. Group mean ± s.e.m. for all analytes are provided in Supplementary Tables 6, 7 and raw data found here: [10.17605/OSF.IO/8QGX9]. In females, several inflammatory mediators, including IL-6, IL-12p-40, and MIP-1α, were significantly increased with aging, but were largely restored to more youthful levels with mAb treatment. Meanwhile, systemic inflammation in old male mice was markedly exacerbated by mAb treatment, as indicated by a marked increase in the majority of measured analytes over age-matched controls. *P < 0.05 for Old mAb versus Old Controls

A rise in pro-inflammatory mediators is a hallmark of aging, thus we evaluated plasma inflammatory markers using a 25-plex immunoassay to determine if these parameters were affected by mAb treatment in male and female mice. Aging in females was characterized by a significant rise in IL-1β, IL-4, IL-5, IL-6, IL-10, IL-12(p40), IL-12(p70), IL-17, CXCL-10, CXCL-1, MIP-1α, MIP-2, and TNFα, but several of these cytokines and chemokines were restored to a more youthful level with mAb treatment (Fig. 5a; Supplementary Table 6; *P* ≤ 0.05). In contrast, only G-CSF, IL-6, and RANTES were elevated in old male plasma, but mAb treatment led to a marked increase in the majority of these markers (Fig. 5b; Supplementary Table 7; *P* ≤ 0.05), demonstrating a clear exacerbation of systemic inflammatory status in male mice.

### L2-Cmu reduces cancer and improves survival in females

**Fig. 6:**
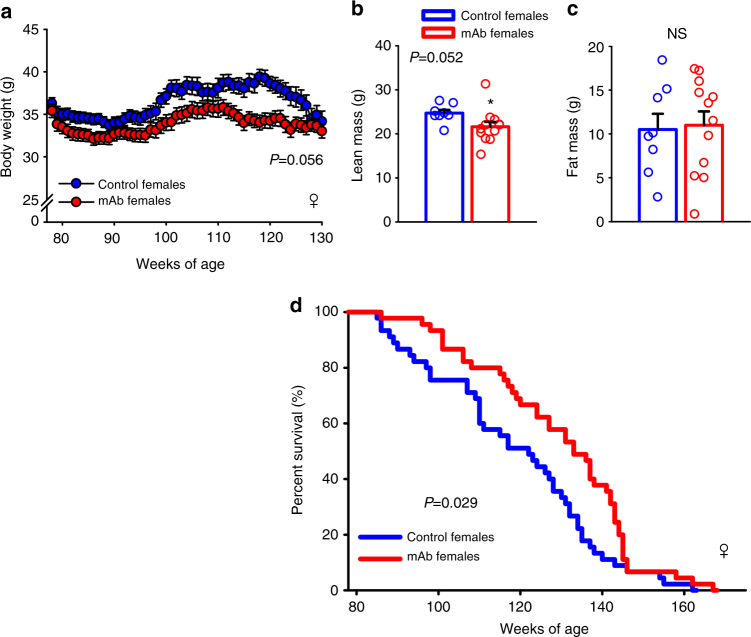
Late-life IGF-1R modulation improves female lifespan. **a** Chronic mAb treatment beginning at 78 wks of age (~18 mo) in CB6F1 female mice tended to cause a slight and persistent reduction in body weight (group effect*, P* = 0.056). **b**, **c** This reduction in body weight was attributed to a reduction in lean mass (*P* = 0.052), rather than adiposity, as assessed by qMR. **d** Importantly, late-life mAb treatment was able to significantly improve mean (*P* = 0.023) and median lifespan (*P* = 0.03), as well as survival after 78 weeks of age (HR = 0.622, *P* = 0.029; *n* = 45 per group), but did not improve maximum lifespan. Details regarding these analyses and related R code can be found in the Statistics section and here: [10.17605/OSF.IO/8QGX9. Bars are means ± s.e.m. Dot plots overlaid on bar graphs represent individual data points. NS not significant. *Significantly different from Controls by independent samples *t-test*, *P* = 0.052

In a 6 mo interim intervention trial with L2-Cmu in older mice, we performed an extensive histopathologic analysis and noted a reduction in endometrial hyperplasia severity (Supplementary Fig. 6a; *P* ≤ 0.05), whereas tumor burden in males tended to be increased (Supplementary Fig. 6b; *P* = 0.07). Furthermore, early indications of potentially improved survival to 24 mo was observed in female mice treated with L2-Cmu (Supplementary Fig. 6c; *P* = 0.41), while male interim survival with L2-Cmu was indistinguishable from Controls (Supplementary Fig. 6d; *P* = 0.77). Thus, to definitively determine if late-life pharmacologic modulation of IGF-1R signaling could improve survival, we performed a longevity study in female mice with lifelong i.p. injections of L2-Cmu once per week, beginning at 18 mo of age until death. As can be observed in this larger cohort (*n* = 45 group), long-term L2-Cmu treatment tended to reduce female body weight (Fig. 6a; *P* = 0.056) and lean body mass (Fig. 6b; *P* = 0.052), with no effect on adiposity (Fig. 6c). It is unclear to what extent the reduction in lean mass is attributable to loss of skeletal muscle mass per se, which comprises only one component of total body lean mass in mice, but the lack of effect on fat mass is consistent with the reported paucity of IGF-1Rs on mature adipocytes. Importantly, late-life L2-Cmu treatment improved mean lifespan (*P* = 0.023) and increased median lifespan by 9% (*P* = 0.03) in females (Fig. 6d), while the risk of death with late-life L2-Cmu treatment was 62.2% (40.7%, 95.2%) of that observed for controls (*P* = 0.029). Furthermore, end-of-life pathology confirmed that deaths due to cancer were significantly reduced, while those attributable to unknown causes were increased by mAb treatment (Table 1; *P* ≤ 0.05), an observation that is consistent with pathologic assessments in caloric-restricted and long-lived GHRKO mice^33^. However, despite improved survival and less cancers with mAb treatment, no significant effect was observed on maximum lifespan (*P* = 0.971).

**Table 1:** Cause of death in female mice

Cause	Con females (*n* = 30)	mAb females (*n* = 20)
Neoplasm	25 (83.3%)	11 (55%)^a^
Lymphoma	12	10
Lymphoma + other tumor	4	1
Adenocarcinoma	5	0
Pituitary adenoma	3	0
Other tumors	1	0
Non-neoplasm	5 (16.7%)	9 (45%)
Glomerulonephritis	1	0
Unknown	4	9^a^

^a^Significantly different from Controls by chi-square test or Fisher’s exact test,

respectively, *P* ≤ 0.05

## Discussion

Across nature, diminished growth factor signaling is linked to improved longevity^12^. Importantly, this relationship is relevant to humans as individuals with exceptional longevity are enriched with functional *IGF-1R* mutations^13^, while low IGF-1 levels predict better survival in female nonagenarians^16^. Given the clear relationship between IGF-1 and aging, we reasoned that IGF-1R mAbs could provide a translational tool to mimic the beneficial effects reported by reduced signaling in this pathway. Here, we provide the first evidence of improved healthspan and age-related survival with a therapeutic mAb by demonstrating that treatment with an IGF-1R antagonist in older mice significantly and preferentially improves several indices of healthspan, reduced death from neoplastic disease, and increased mean and median lifespan in females. Similar to rapamycin^31^, these effects were achieved even though not initiated until later in life, which we reasoned to be a safer therapeutic window for IGF-1R modulation than younger ages^34^.

In agreement with previous evidence from genetic models, these data show that chronic modulation of IGF-1R signaling may be most well suited for targeting aging in females^6–8^, rather than males. Such an indication is unique from most other drugs and compounds identified by the NIA-supported Intervention Testing Program (ITP) to improve lifespan, as acarbose, 17-α-estradiol, nondihydroguaiaretic acid (NDGA), and protandim, all preferentially improve male lifespan^35,36^. The potential explanation for sex differences in the IGF-1 signaling pathway on aging as well as response to other age-delaying interventions is unclear, but unique interactions of candidate pathways and targets with sex hormones could explain in part these disparate effects. Likewise, sex differences in the way some of these drugs are absorbed and metabolized has been noted^37^, though we did not observe any differences in L2-Cmu PK or exposure levels between male and female animals.

Beyond the demonstration that pharmacologic blockade of IGF-1R signaling can favorably impact lifespan, these data also provide new insights into temporal and metabolic aspects regarding this pathway and aging. As previously mentioned, examples of longevity resulting from disruption of the GH/IGF-1 and/or insulin signaling pathway are mainly derived from early life manipulations^6,17,18^, whereas brief exposure to GH (and IGF-1) treatment early in development is sufficient to partially abrogate the exceptional longevity of Ames Dwarf mice^20^. In both male and female GHRKO mice, disruption of the GH/IGF-1 axis from birth results in mice that are remarkably long lived, but temporal disruption of this pathway in young adults (aGHRKO mice) improves lifespan only in females^17^, an observation which is consistent with the beneficial effects of targeting IGF-1R signaling post-development on female healthspan and aging in this study. Interestingly, adult mice with modest isolated elevations in GH and IGF-1 levels have greater lean mass, improved lipid oxidation and glucose clearance^38^, while specific, temporal reductions in GH and IGF-1 in adults have been linked to deleterious metabolic effects, including impaired hepatic insulin action^38^, and reduced β-cell mass and function^39^. Thus, specific targeting of IGF-1R signaling post development may prove to be the best approach to achieve the benefits of low growth signals on healthspan and aging, without the potential unwanted side effects of combined adult-onset GH/IGF-1 deficiency.

While identifying the optimal therapeutic window will require further investigation, we reasoned that starting later in life, a time in which function of the somatotropic axis is diminished, would be the best approach. Indeed, a rise in IGF-1 levels have been observed in multiple studies of IGF-1R blockade, which is attributed to the reduction in IGF-1-mediated inhibition of GH secretion^26,40^. For instance, administering IGF-1R mAbs in cancer trials was reported to result in increased adverse events in younger populations^34^, including insulin resistance, possibly due in part to disruption of the somatotropic axis and associated deleterious effects of excess GH^41,42^. However, we did not observe significant differences in circulating IGF-1 levels with mAb treatment in older mice, though levels tended to be higher in mAb-treated females, but glucose homeostasis was not adversely affected in either sex, suggesting minimal disruption of the somatotropic axis in this older cohort of mice.

Interestingly, a recent study examining the time-dependent effect of low IGF-1 levels on aging outcomes observed that a targeted reduction in liver-derived IGF-1 early (10 d or 5 mo) or later in life (15 mo) improved aspects of lifespan in females, though these effects were most pronounced when IGF-1 was reduced at 5 mo old^19^. In contrast, male lifespan tended to be reduced by early-life IGF-1 deficiency, which is in agreement with a prior study in LID mice^41^. Therefore, these findings further support the observation that low IGF-1 preferentially benefits females, and suggest that a potentially broad therapeutic window to target IGF-1R signaling may exist in females, with greater benefit possible by initiating at even younger ages than implemented in this study. Further optimization of the timing, dose, and duration of IGF-1R mAb treatment is warranted in order to maximize benefits while minimizing risks, particularly given recent evidence that transient administration of other drugs can have persistent benefits on age-related outcomes^43^.

Beyond the observed increase in female lifespan, we show that pharmacologic blockade of IGF-1R signaling preserves, to various degrees, many aspects of healthspan, including neuromuscular function and endurance, and attenuates the age-related increase in diastolic dysfunction, systemic inflammation, and neoplasias in females. The observed sex dimorphism in inflammatory cytokines was a particularly intriguing and unexpected observation, which beyond serving as a marker of aging status, could be reflective of the relative increase and decrease in neoplasms, documented in male and female mice, respectively. However, it is not clear what contributed specifically to this effect on inflammation, though increasing evidence suggests that IGF-1 signaling is critical for maintaining immune cell homeostasis^44^, but the prospects of sex differences in the requirement for IGF-1 signaling in these cells is not yet known and will require further study.

While the beneficial effects of low IGF-1 action on aging seems well supported by data in model systems^6,7^ and unique human populations^13,16^, a broader examination of the literature suggests that a more nuanced, complex, and controversial relationship exists between IGF-1, aging, and disease, which is indicative of the pleiotropic actions attributed to IGF-1 in cells and tissues^12,19^. Certainly, an increase in IGF-1 action is associated with greater risk for neoplastic disease, a link further supported by this study and others targeting IGF-1Rs, but IGF-1 has been paradoxically associated with protection against osteoporosis, type 2 diabetes, cerebrovascular and cognitive decline in pre-clinical studies, as well as in some human epidemiologic studies^12^. Therefore, both temporal and spatial considerations of IGF-1R modulation will need to be considered in future trials with these drugs in order to clearly define the benefits and potential risks associated with this treatment, and guide development of strategies that allow for safe and effective long-term human use. Finally, while the focus of this investigation has centered on effects of inhibiting IGF-1 binding to the IGF-1R and HybridRs, L2-Cmu also interferes with IGF-2 binding to these receptors. However, as opposed to adult humans, where IGF-2 levels are substantial, systemic IGF-2 levels are undetectable in adult rodents. Thus, the inability to account for inhibition of IGF-2 binding to the IGF-1R is an important limitation in this study, which should be considered when assessing outcomes from human trials using IGF-1R mAbs.

In summary, these data provide evidence that long-term IGF-1R blockade with mAbs is feasible, well tolerated, and can recapitulate effects observed with genetic disruption of IGF-1R signaling on lifespan. As IGF-1R mAbs are already in clinical trials, these observations warrant further exploration into the possible development of these drugs for safe and effective long-term use in humans as a strategy to delay at least some manifestations of aging. Finally, these observations provide a unique example of an intervention which appears to favor female healthspan and lifespan, thereby reinforcing the need for considering sex differences in devising therapeutic strategies to treat aging and its diseases.

## Methods

### Animals

Young (4 mo) and old (18 mo) male and female CB6F1 mice were obtained from the NIA Aged Rodent Colony. All animals were housed at standard temperature (~22°C) and humidity-controlled conditions under a 14L:10D photoperiod and provided ad libitum access to water and a low-fat-purified diet upon arrival (10% calories from fat D12450H Research Diets Inc). In vivo L2-Cmu validation studies were performed in 4–5-mo-old C57BL/6J male mice (Jackson Labs, Bar Harbor, ME). All experiments were approved by the Institutional Animal Care and Use Committee at the Albert Einstein College of Medicine (Protocol #20140107).

### L2-Cmu development and validation

To target IGF-1R action in mice, we utilized the mAb, L2-Cmu (Amgen Inc, Thousand Oaks, CA), which is a murinized IgG1 version of the fully human L2-C mAb previously reported by Calzone et al.^25^. Specifically, L2-Cmu was engineered as a human/murine chimera (i.e., reverse chimeric antibody), such that the complementary-determining regions (CDRs) were engineered into a new framework in which the entirety of the variable regions are human and the constant regions are mouse. Unlike the human IgG1 constant region of L2-C, which might induce effector functions, the murine IgG1 constant region would not be expected to elicit effector function activity. Validation of L2-Cmu was confirmed by Biacore analysis and in murine fibroblasts (NIH-3T3) in vitro. For NIH-3T3 experiments, cells were grown in DMEM plus 10% fetal bovine serum (Invitrogen). At *t* = −4 h, cells were serum starved and at *t* = −1 h, pre-treated with vehicle, control IgG1, or L2-Cmu (100 µg/mL). Vehicle IGF-1 (5 nM) or IGF-2 (20 nM) was then added to the media for 2 min and cells were then rapidly lysed in ice-cold buffer to assess receptor activation as described below.

For in vivo validation of L2-Cmu blockade, C57BL/6J mice were sedated with 2% isoflurane for surgical placement of an indwelling catheter into the right internal jugular vein^45^. Animals were then assigned to receive either an initial i.p. injection of L2-Cmu (*n* = 6) at a dose of 20 mg/kg or vehicle (*n* = 9) 5 days after surgery. On day 7, animals were fasted at 0900 h and then received a second i.p. injection of either L2-Cmu or vehicle. Approximately 4 h later, animals were assigned to receive either a peripheral saline (Control *n* = 4) or IGF-1 i.v. infusion (5 µg total dose) over 1 min at a rate of 100 µL/min, resulting in three experimental groups: Control (vehicle + saline; *n* = 4), IGF-1 (vehicle + IGF-1; *n* = 5), and IGF-1 plus L2-Cmu (*n* = 5 for lung and *n* = 6 for heart). Animals were then quickly sacrificed 1 min after completion of the infusion, and tissues rapidly excised, snap-frozen, and stored at −80 °C for receptor activation assays.

### Experimental design and treatment

CB6F1 mice were assigned to receive either weekly i.p. injections of vehicle or L2-Cmu (20 mg/kg) once per week and were monitored for interim survival to 24 mo of age (*n* = 24–38 per group) and sacrificed for blood, tissue, and histopathologic analysis. For hormone and metabolite analyses, animals were fasted at 0800 h, and trunk blood was routinely collected 4 h later at 1200 h by decapitation without anesthesia, and plasma was separated from red cells by centrifugation (1500 × *g* × 4 °C × 15 min) and stored at −80 °C. For the survival study, animals were treated with i.p. vehicle or L2-Cmu until natural death (females only; *n* = 45 group). Animals deemed severely moribund and anticipated to not survive another 24 h were immediately euthanized and this was considered the time of death. In addition, histopathology was conducted at 24 mo of age or end of life, as described below.

### L2-Cmu pharmacokinetic and chronic exposure assessments

For PK studies, CB6F1 mice (*n* = 4–5 per sex, per timepoint) were injected i.p. with L2-Cmu (20 mg/kg) and sacrificed at either 6 h, 24 h, 3 days or 7 days later, and plasma was isolated from whole blood. A baseline group was included, which was injected with vehicle 6 h prior to sacrifice. For determining chronic exposure levels, plasma was obtained 48 h following dose 24 of vehicle or L2-Cmu by i.p. injection in male Con (*n* = 9) and L2-Cmu (*n* = 34) and female Con (*n* = 9) and L2-Cmu (*n* = 30) mice. L2-Cmu plasma concentrations were measured using an ELISA method with an LLOQ of 0.1 µg/mL and assay range of 0.1–20 µg/mL. Briefly, the ELISA plate (Corning 3690) was coated with murine anti-AMG 479, clone 3 at 2 µg/mL in 1× PBS and incubated overnight at 4 °C. Standards and quality controls were prepared by spiking L2-Cmu into mouse plasma. The ELISA plate was washed and blocked with I-BlockTM (Applied Biosystems) buffer (1× PBS + 0.2% I-Block + 0.05% Tween20) for 1 h at room temperature. Mouse plasma was used as a diluent for samples when necessary. The standards and samples were then diluted 1:4 in assay buffer (I-Block buffer + 5% BSA). The diluted standards and samples were loaded into the ELISA plate and allowed to incubate for 1.5 h. After a wash step, a horseradish peroxidase (HRP) conjugated murine anti-AMG 479, clone B35 at 400 ng/mL was added to the plate and incubated for 1.5 h. After a final wash step, a tetramethylbenzidine (TMB) peroxidase substrate solution (KPL Inc.) was added to each well and absorbance of the developed color was measured by a colorimetric plate reader (Molecular Device).

### Metabolic phenotyping

Body weight was monitored on a weekly basis and body composition was assessed at 3 mo intervals by qMR (ECHO MRS; Echo Medical Systems). Glucose tolerance tests (GTT) and insulin tolerance tests (ITT) were performed (*n* = 12 per group, per sex) to assess glucose metabolism and insulin sensitivity ^46^. For GTTs, animals were fasted for 4 h and a baseline blood glucose measurement was made prior to administering a 2 mg/kg i.p. glucose injection. Blood glucose was subsequently monitored at 15, 30, 60, 90, and 120 min post injection with a glucose meter (Bayer Contour). ITTs were performed in random-fed mice, early in their light cycle (~0700h–0800h)^46^. Following a baseline glucose measurement, mice were injected. i.p. with 0.75 U/kg insulin and blood glucose was measured at 15, 30, 45, and 60 min later.

Energy expenditure, substrate utilization, food intake, and spontaneous activity were determined^45,47^, based upon O_2_ consumption and CO_2_ production, using a Mouse CLAMS System (Columbus Instruments, Columbus, OH). In brief, animals (*n* = 8 per group, per sex) were placed into individual cages at their standard temperature and photoperiod and allowed to acclimate for at least 72 h prior to the experiment, and data were collected over a 24 h period.

### Functional healthspan assessments

At 23–24 mo of age, motor coordination, strength, and endurance were evaluated in mice using a battery of healthspan assessments. Neuromuscular function was determined via balance beam in females [Young (*n* = 8), Old Con (*n* = 12), and Old mAb (*n* = 15)] and males [Young (*n* = 8), Old Con (*n* = 9), and Old mAb (*n* = 9)]. In brief, animals were first familiarized with walking across a 4 ft plank prior to testing three round beams of increasing difficulty (1″ easy; 0.75″ medium, 0.5″ difficult), with light and food cues as motivation to cross, and the number of slips were counted while traversing the beam^48^. Forelimb grip strength was determined by allowing animals to clasp a suspended wire and the time to release was recorded in females [Young (*n* = 8), Old Con (*n* = 12), and Old mAb (*n* = 15)] and males [Young (*n* = 8), Old Con (*n* = 10), and Old mAb (*n* = 9)]. Exercise capacity was determined by a single maximal exercise test to voluntary fatigue on a motorized treadmill (Exer 3/6, Columbus Instruments) in females [Young (*n* = 10), Old Con (*n* = 17), and Old mAb (*n* = 22)] and males [Young (*n* = 10), Old Con (*n* = 9), and Old mAb (*n* = 9)]. Mice were first familiarized to the treadmill for three non-consecutive days for 5 min at a walking speed (8 m/min). Animals were then challenged with a graduated fatigue test, beginning at 10 m/min and 4% grade for 3 min, and increasing in speed by 2 m/min every 2 min to a max speed of 16 m/min until exhaustion.

### Cardiovascular phenotyping

Systolic and diastolic function was evaluated following 5–6 mo of treatment^19,42^. In brief, mouse electrocardiography was measured in females [Young (*n* = 6), Old Con (*n* = 8), and Old mAb (*n* = 7)] and males [Young (*n* = 5), Old Con (*n* = 6), and Old mAb (*n* = 5)], using a Visual Sonic Vevo2100 imaging system (FUJIFILM VisualSonics Inc, Toronto, ON). Cardiac left ventricular dimensions were obtained under M-mode, and left ventricle ejection fraction (EF) and fractional shortening (FS) were calculated accordingly. Left ventricular diastolic function, presented as the E/A ratio, was generated based on transmitral blood flow measured under Color Doppler mode. At sacrifice, heart tissue was immediately harvested, and the heart was perfused and fixed with 10% neutral-buffered formalin (NBF) for 24 h. Following fixation, hearts were embedded in paraffin and 5 μm sections were mounted onto treated slides, and stained with hematoxylin and Eosin (H&E) and co-stained with Masson’s trichrome. Tissue fibrosis was quantified in females [Young (*n* = 4), Old Con (*n* = 5), and Old mAb (*n* = 5)] and males [Young (*n* = 8), Old Con (*n* = 8), and Old mAb (*n* = 8)], by counting blue stained interstitial collagen within three random fields using Image J and values were averaged.

### Metabolomic analysis

We used Biocrates AbsoluteIDQ p180 kit to analyze cardiac metabolites from female mice [Young (*n* = 7), Old Con (*n* = 8), and Old mAb (*n* = 7)] with UPLC-MS/MS Xevo TQ, Waters, Pittsburgh, PA, USA) in the Einstein Stable Isotope and Metabolomics Core, according to the manufacturer’s instructions (BIOCRATES Life Sciences AG, Innsbruck, Austria). Heart tissue samples were weighed, homogenized with 8 times of 2.5 mM ammonium acetate in methanol, and 20 µL of the extraction from each sample was used for the assay. A pooled quality control (QC) sample was added to the sample list. This QC sample was plated at different positions on the 96-well plate and injected six times to calculate the coefficient of variation (CV) for data quality control, and undetectable metabolites were excluded from the analysis. The dataset was then imported into R software [R version 3.4.2] and normalized using log transformation for multivariate analysis, unsupervised principle component analysis (PCA), and partial least squares-discriminant analysis (PLS-DA).

### Histopathology

Complete histopathology was performed in 24-mo-old female [Old Con (*n* = 16), and Old mAb (*n* = 16)] and male mice [Old Con (*n* = 15), and Old mAb (*n* = 17)] following 6 mo of mAb treatment, as well as in female mice at death from the longevity study [Old Con (*n* = 30), and Old mAb (*n* = 20)]. In brief, a gross evaluation was conducted when possible and then a complete necropsy was performed. Tissues were infiltrated with paraffin and H&E sections were obtained. Slides were shipped to the University of Texas at San Antonio Pathology Core and evaluated by two pathologists who were blinded to the experimental groups (Y.I. and G.B.H.). Diagnosis of each histopathological change was made using histological classifications for aging mice^33,49,50^. In brief, a list of lesions was compiled for each mouse that included both neoplastic and non-neoplastic diseases. Based on these histopathological data, tumor burden, disease burden, and severity of lesions in each mouse were assessed.

Tumor burden was calculated as the sum of the different types of tumors in each mouse. The disease burden was similarly calculated as the sum of the histopathological changes in a mouse and severity of neoplastic and renal lesions was assessed using an established grading system. The probable cause of death was determined independently by both pathologists based on the severity of the pathology found at necropsy. In cases with neoplastic lesions, mice with Grade 3 or 4 lesions were categorized as death by neoplastic disease. In more than 90% of cases, there was agreement by the two pathologists. In cases where the two pathologists did not agree or where disease did not appear severe enough, the cause of death was categorized as unknown.

### Blood measures

Clinical blood chemistries and related measures were determined in whole blood and serum of female [Old Con (*n* = 7) and Old mAb (*n* = 8)] and male mice [Old Con (*n* = 7) and Old mAb (*n* = 8)] by Antech Diagnostics (New Hyde Park, NY). Basal insulin was measured in plasma from female [Young (*n* = 16), Old Con (*n* = 26), and Old mAb (*n* = 25)] and male mice [Young (*n* = 17), Old Con (*n* = 32), and Old mAb (*n* = 28)] using a bead-based assay for mouse insulin (EMD Millipore, Inc) and detection was performed on a Bio-Plex MAGPIX Multiplex Reader (Biorad Inc., Hercules, CA). Plasma IGF-1 levels were measured using the Mouse/Rat IGF-1 Quantikine ELISA Kit (MG100; R&D Systems) in plasma from female [Young (*n* = 8), Old Con (*n* = 16), and Old mAb (*n* = 15)] and male mice [Young (*n* = 8), Old Con (*n* = 15), and Old mAb (*n* = 16)]. In addition, a MAGPIX Multiplex Reader was used to measure 25 inflammatory mediators simultaneously in plasma from female [Young (*n* = 8), Old Con (*n* = 15), and Old mAb (*n* = 16)] and male mice [Young (*n* = 8), Old Con (*n* = 14), and Old mAb (*n* = 16)], including: G-CSF, GM-CSF, IFN-γ, IL-1α, IL-1β, IL-2, IL-4, IL-5, IL-6, IL-7, IL-9, IL-10, IL-12 (p40), IL-12 (p70), IL-13, IL-15, IL-17, CXCL-10, CXCL-1, MCP-1, MIP-1α, MIP-1β, MIP-2, RANTES, and TNF-α (MCYTOMAG-70K-PMX; EMD Millipore, Billerica, MA).

### Protein isolation and western blotting

For standard western blotting, tissues were lysed in RIPA buffer and extracted protein content was determined using the BCA protein assay (Sigma, St. Louis, Mo) with BSA as a standard ^45,51^. In brief, protein was separated on Bis Tris Stain-Free gels (4–20%) and electrophoresed at 120 V constant for 90 min (*n* = 8 per group, per sex)^45,51^. Prior to transfer, stain-free gels were imaged on a Biorad Chemidoc MP Imaging System (Biorad, Hercules, CA) to confirm equal protein load, and were then wet transferred onto PVDF membranes at 100 V constant for 1 h and equal transfer was routinely confirmed by Ponceau S stain. Following block in 5% milk or BSA, membranes were incubated with an appropriate primary antibody from Cell Signaling (Danvers, MA) against pAkt^Ser473^ (1:1000; #4060), total Akt (1:1000; #4691), p-p44/42MAPK^Thr202/Tyr204^ (1:1000; #9101) total p44/42 MAPK (1:1000; #4695), pS6 (1:1000; #5364), Total S6 (1:1000; #2217), total IGF-1R (1:1000; #9750), and InsRβ (1:1000; #3025) overnight at 4 °C. Following a 1 h incubation with the appropriate secondary antibody, Clarity Western ECL Substrate was applied to the membrane and bands were visualized using a Biorad Chemidoc MP to first pixel saturation and densitometry performed using Image Lab (Biorad, Hercules, CA). Complete, uncropped western blot images from figures are provided in Supplementary Fig. 7.

### Immunoprecipitation

For immunoprecipitation assays, NIH-3T3 cell or tissue  protein, respectively, was extracted with a non-denaturing cell or tissue  extraction buffer (Invitrogen/ThermoFisher, Carlsbad, CA). Immunoprecipitation was then performed using the Catch and Release Immunoprecipitation Kit (EMD Millipore), according to the manufacturer’s instructions with 250 µg of total protein from cells or 400 µg from tissues, and 1 µg of an anti-IGF-1R antibody (#9750, Cell Signaling). Following electrophoresis and transfer, membranes were blotted with either a pTyr antibody (1:1000; #8954, Cell Signaling) for IGF-1R activation, or an anti-IGF-1R antibody (#9750, Cell Signaling) for total levels. For IGF-1R/InsR HybridR activation, IGF-1R immunoprecipitates were probed with an antibody against the InsR specific pTyr^1334^ residue (#44809G, Invitrogen/ThermoFisher)^52^, and total HybridR determined by immunoblotting with an Anti-InsRβ antibody (#3025).

### Statistics

All values are presented as means ± SE. Longitudinal data were assessed using a linear mixed effects model, with group as a categorical variable and time as a continuous variable, and cross sectional data were assessed either by independent samples *t*-test or one-way ANOVA. When a significant effect in the model was observed, planned two-group contrasts (Tukey Honest Significant Difference [HSD] method) were applied. Normality assumption was examined prior to analysis and data were log transformed when appropriate to ensure normality of distribution. When the normality assumption was uncertain, as was determined for plasma cytokines, non-parametric Kruskal–Wallis test and Mann–Whitney *U* test were used. The total frequency and grade of pathologic lesions were compared between genotypes using a chi-square test. When the expected frequencies were too small in any group (*n* < 5), the Fisher’s exact test was used instead. Survival analysis was performed by the UAB Comparative Data Analytics Core. As animals were obtained in six separate batches due to operational limitations and grouped in cages, the survival curves were plotted using the Kaplan–Meier method. The treatment effect was then evaluated using Cox proportional hazard regression, with cage assignment included as a random effect and batch as a covariate, using statistical software R (version 3.4.1) with package “coxme”. For testing the difference in mean and median lifespan between groups, we used linear mixed model with linear quantile mixed-effects model, which included cage assignment as a random effect and batch as a covariate, respectively, using package “nlme” and “lgmm” in statistical software R. Effects on maximum lifespan were determined by setting the threshold for lifespan to the 90th percentile for both groups combined^53^. All other statistical analyses were performed using either SPSS (SPSS Inc, Chicago, IL) or JMP software version 9 (SAS Institute Inc., Cary, NC). For metabolites, a nominal *P* ≤ 0.05 and FDR ≤ 0.05 was considered significant, and a *P* ≤ 0.05 and FDR ≤ 0.15 considered marginally significant. For all other analyses, *P* ≤ 0.05 was considered statistically significant.

### Data availability

All data supporting the findings of this study are included in this published article and its Supplementary Information files, and are available from the corresponding author upon request. Datasets for survival, metabolomics, and cytokines are available here: [10.17605/OSF.IO/8QGX9]

### Code availability

The R code for survival and metabolomic analyses are available here: [10.17605/OSF.IO/8QGX9]

## Electronic supplementary material

Supplementary Information
