# Supplementary material for: Late-life targeting of the IGF-1 receptor improves healthspan and lifespan in female mice
Source: Nat Commun. 2018 Jun 19;9:2394. doi: 10.1038/s41467-018-04805-5 (PMC6008442; doi:10.1038/s41467-018-04805-5)

SUPPLEMENTARY INFORMATION

Late-life targeting of the IGF-1 receptor improves healthspan and lifespan in female mice

Mao et al

Supplementary Table 1.

Biacore measurement of L2-Cmu monoclonal antibody binding.

mAb	Kd (nM)	IGF-1 Ki (nM)	IGF-1 Max (%)	IGF-2 Ki (nM)	IGF-2 Max (%)
L2-Cmu	0.30	3.3	99	3.3	99
αIR3	0.33	>1000	31	>1000	NI

Biacore measurement of antibody binding to murine IGF-1R(ECD)-C3-mFc was measured in parallel by the kinetic method using L2-Cmu and the anti-IGF-1R mAb (clone αIR3). A precise value was not obtained due to limitations in the amount of mu IGF-1R(ECD)-muFc. Ligand blocking measured in the IGEN format with murine IGF-1R(ECD)-C3-mFc and human Ru labeled IGF-1 and IGF-2. NI = No inhibition.

Supplementary Table 2.

Female red and white blood cell counts following 6 mo mAb Treatment.

Parameter	Con Females (*n*=5-7)	mAb Females (*n*=8)	p-value	Reference Range
RBC 10^3/uL	9.8 ± 0.3	8.1 ± 0.2	0.005	5.5-10.5
Hemoglobin g/dL	15.7 ± 0.3	13.8 ± 0.3	0.008	13.0-15.0
Hematocrit %	49.8 ± 1.7	42.4 ± 1.9	0.048	33-50
MCV Mean Corpuscular Volume fL	50.3 ± 0.4	52.1 ± 1.5	0.39	
MCH mean corpuscular hemoglobin pg	16.0 ± 0.5	17.0 ± 0.3	0.13	
MCHC mean corpuscular hemoglobin concentration g/dL	31.9±1.1	32.8 ± 1.4	0.66	
Platelet Count 10^3/uL	849.3±56.7	901.6±139.2	0.82	
WBC	6.0±0.4‡	3.6 ± 0.7	0.02	5.5-10.5
Neutrophils /uL	1081 ± 69.7‡	635.8±106.4	0.06	
Neutrophils %	19.8 ± 7.9%‡	20.4 ± 4.3	0.21	
Bands	0	0		
Lymphocytes /uL	4166 ± 358‡	2781 ± 610	0.04	
Lymphocytes %	75 ± 2	73 ± 6	0.82	
Monocytes /uL	464 ± 248	123 ± 24	0.17	
Monocytes %	3.6 ± 0.8	4.7 ± 1.6	0.56	
Eosinophils /uL	79.4 ± 32.7	52.9 ± 29.7	0.55	
Eosinophils %	1.6 ± 0.7	2.2 ± 1.3	0.68	
Basophils /uL	0	8.1 ± 7.6	0.34	
Basophils %	0	0.13 ± 0.13	0.37	

Data are means± s.e.m.

‡Two statistical outliers were removed from this group.

Supplementary Table 3.

Female blood chemistries following 6 mo mAb Treatment.

Parameter	Con Females (*n*=7)	mAb Females (*n*=8)	p-value	Reference Range
Total Protein g/dL	5.2 ±0.1	5.3 ±0.1	0.13	4.5-6.5
Albumin g/dL	2.8 ±0.1	2.8 ±0.1	0.84	2.4-4.4
Globulin g/dL	2.4 ±0.1	2.6 ±0.1	0.12	2.4-4.4
AST U/L	163.4 ±23.1	142.0 ± 18.2	0.46	10-45
ALT U/L	48.6 ±10.9	31.0 ±3.4	0.13	10-35
Alk Phosphatase U/L	86.1 ±8.4	101.5 ±16.9	0.43	15-45
Total Bilirubin mg/dL	0.17 ±0.18	0.20 ±0.04	0.34	0-1
Urea Nitrogen mg/dL	17.0 ±0.9	15.8 ±1.2	0.44	9-30
Creatinine mg/dL	0.19 ±0.01	0.20 ±0.01	0.30	0.4-1.0
Phosphorus mg/dL	7.5 ±0.3	6.4 ±0.3	0.02	4.2-8.5
Glucose mg/dL	150.6 ±10.5	160.0 ±6.2	0.48	60-125
Calcium mg/dL	9.2 ±0.2	8.2 ±0.6	0.16	8-12
Sodium mEq/L	148.7 ±0.9	146.9 ±0.6	0.10	140-160
Potassium mEq/L	5.6 ± 0.3	5.6 ±0.4	0.97	4.3-5.8
Na/K Ratio	26.7 ±1.2	26.8 ±1.6	0.99	
Chloride mEq/L	112.1 ±1.2	111.8 ±0.6	0.76	90-110
Cholesterol Mg/dL	109.6 ±6.0	106.9 ±4.2	0.71	50-250
CPK U/L	680.3 ±243.8	890.9 ±178.4	0.48	

Data are means± s.e.m.

Supplementary Table 4.

Male red and white blood cell counts following 6 mo mAb Treatment.

Parameter	Con Males (*n*=7)	mAb Males (*n*=8)	p-value	Reference Range
RBC 10^3/uL	8.0 ± 0.8	7.67 ± 0.2	0.79	5.5-10.5
Hemoglobin g/dL	13.1 ± 1.3	12.89 ± 0.3	0.93	13.0-15.0
Hematocrit %	41.0 ± 4.3	40.14 ± 1.9	0.90	33-50
MCV Mean Corpuscular Volume fL	50.9 ± 0.9	51.43 ± 1.5	0.67	
MCH mean corpuscular hemoglobin pg	16.3 ± 0.5	17.15 ± 0.3	0.25	
MCHC mean corpuscular hemoglobin concentration g/dL	32.0 ± 1.0	33.4 ± 1.4	0.34	
Platelet Count 10^3/uL	1040.7 ± 182.9	1011 ± 166.8	0.91	
WBC	5.1 ± 0.8‡	5.3 ± 1.0	0.91	5.5-10.5
Neutrophils /uL	1207 ± 303‡	1340 ± 357	0.79	
Neutrophils %	21.8 ± 3.2‡	28.6 ± 5.3	0.41	
Bands	0	0		
Lymphocytes /uL	3676 ± 484‡	3726± 837	0.96	
Lymphocytes %	73.5 ± 6.2‡	66.8 ± 5.2	0.42	
Monocytes /uL	164.7 ± 55.5	122.5 ± 45.1	0.55	
Monocytes %	2.1 ± 0.3	2.3 ± 0.5	0.86	
Eosinophils /uL	183.1 ± 76.7	93.0 ± 42.2	0.30	
Eosinophils %	2.4 ± 1.0	3.2 ± 1.2	0.65	
Basophils /uL	0	0		
Basophils %	0	0		

Data are means± s.e.m.

‡One statistical outlier was removed from this group.

Supplementary Table 5.

Male blood chemistries following 6 mo mAb Treatment.

Parameter	Con Males (*n*=7)	mAb Males (*n*=8)	p-value	Reference Range
Total Protein g/dL	4.7 ±0.2	5.8 ±0.1	0.002	4.5-6.5
Albumin g/dL	2.4 ±0.1	2.6 ±0.1	0.34	2.4-4.4
Globulin g/dL	2.3 ±0.1	3.1 ±0.1	<0.001	2.4-4.4
AST U/L	108.3 ±21.5	83.2 ±8.4	0.34	10-45
ALT U/L	20.7 ±3.6	33.9 ±3.4	0.02	10-35
Alk Phosphatase U/L	42.6 ±5.5	47.7 ±5.4	0.53	15-45
Total Bilirubin mg/dL	0.17 ±0.03	0.11 ± 0.01	0.10	0-1
Urea Nitrogen mg/dL	20.3 ± 1.4	18.3 ±1.2	0.31	9-30
Creatinine mg/dL	0.21 ±0.01	0.16 ±0.02	0.04	0.4-1.0
Phosphorus mg/dL	7.3 ±0.5	7.5 ±0.3	0.74	4.2-8.5
Glucose mg/dL	154.1 ±21.5	136.4 ±6.9	0.45	60-125
Calcium mg/dL	9.3 ±0.1	9.1 ±0.2	0.47	8-12
Sodium mEq/L	152.3 ±0.7	150.7 ±0.8	0.19	140-160
Potassium mEq/L	5.6 ±0.3	5.9 ±0.1	0.47	4.3-5.8
Na/K Ratio	27.6 ±1.5	25.7 ±0.8	0.31	
Chloride mEq/L	112.0 ±1.1	109.8 ±0.5	0.11	90-110
Cholesterol Mg/dL	123.6 ±15.3	139.7 ±19.5	0.56	50-250
CPK U/L	827.2 ±244.0	652.5 ±95.6	0.55	

Data are means± s.e.m.

Supplementary Table 6.

Inflammatory cytokines and chemokines in female mice.

**Analyte (pg/mL)**	**Young (*n*=8)**	**Old Con (*n*=15)**	**Old mAb (*n*=16)**
G-CSF	283.2±31.5[a]	917.9±437.7[ab]	265.4±117.5[b]
GM-CSF	5.5±0.0	25.6±6.8	16.5±7.8
IFNγ	0.6±0.0	6.3±2.4	7.0±6.5
IL-1α	84.7±14.4	107.3±12.7	138.4±45.5
IL-1β	2.7±0.0[a]	707.5±636.4[b]	69.6±60.9[ab]
IL-2	0.5±0.0	6.1±2.4	1.3±0.8
IL-4	0.2±0.0[a]	213.1±95.6[b]	24.5±24.3[a]
IL-5	4.4±2.4[a]	345.1±200.6[b]	57.1±53.5[a]
IL-6	0.6±0.0[a]	1134.8±560.0[b]	38.3±36.0[a]
IL-7	0.7±0.0[a]	46.2±39.4[ab]	151.0±57.9[b]
IL-9	ND	ND	ND
IL-10	1.0±0.0[a]	2284.4±1191.0[b]	81.6±52.1[b]
IL-12(p40)	2.5±0.6[a]	41.9±20.0[b]	5.8±2.1[a]
IL-12(p70)	2.4±0.0[a]	2485.2±1190.0[b]	139.8±107.0[ab]
IL-13	62.1±8.6	181.3±67.5	69.2±19.0
IL-15	3.7±0.0[a]	300.2±282.9[ab]	1035.5±413.5[b]
IL-17	0.25±0.0[a]	549.1±251.6[b]	15.0±13.0[ab]
CXCL-10	99.3±8.9[a]	209.6±78.2[b]	327.1±202.3[ab]
CXCL-1	27.5±5.8[a]	67.8±14.3[b]	51.4±19.8[a]
MCP-1	3.4±0.0[a]	248.3±187.0[b]	69.4±38.6[ab]
MIP-1α	28.2±10.2	63.8±13.5 [b]	14.5±7.5[a]
MIP-1β	6.0±0.0	20.5±8.9	48.6±27.8
MIP-2	39.8±5.9[a]	157.0±43.8[b]	63.2±13.8[a]
RANTES	5.2±1.6	775.8±474.8	35.6±21.0
TNFα	1.2±0.0[a]	66.1±53.2[b]	13.9±7.4[ab]

Data are means± s.e.m. Corresponding log-transformed data Heatmap is shown in Figure 5a. Data were analyzed by the Kruskal-Wallis procedure and the Mann-Whitney U test when appropriate. Any value below the lower limit of detection of the assay was replaced by the minimal detectable concentration (MOD)/√2 for the specific analyte, and these values were ranked as a tie for purposes of the statistical analysis. Different letters denote a significant difference between groups, $P \leq 0.05$.

Supplementary Table 7.

Inflammatory cytokines and chemokines in male mice.

Analyte (pg/mL)	Young (n=8)	Old Con (n=14)	Old mAb (n=16)
G-CSF	179.8±21.0[a]	128.9±48.4[b]	174.2±73.4[b]
GM-CSF	6.5±1.0[a]	9.1±2.4[a]	37.4±7.7[b]
IFNγ	3.7±1.8[a]	6.4±2.9[a]	36.9±7.8[b]
IL-1α	31.2±7.2[a]	32.0±6.0[a]	116.2±50.9[b]
IL-1β	2.2±0.3[a]	8.8±6.3[a]	96.0±79.2[b]
IL-2	0.5±0.0[a]	0.7±0.2[ab]	7.3±3.3[b]
IL-4	0.2±0.0[a]	2.8±2.5[a]	16.0±11.1[b]
IL-5	1.0±0.2[a]	6.6±5.1[a]	41.3±18.8[b]
IL-6	0.6±0.1[a]	4.5±1.9[b]	33.9±15.3[b]
IL-7	2.1±0.5[a]	163.6±189.6[a]	218.3±202.6[b]
IL-9	76.5±18.4	57.5±12.0	736.8±665.0
IL-10	1.0±0.3[a]	3.4±1.7[a]	58.5±29.3[b]
IL-12(p40)	7.1±1.2[a]	6.7±2.4[a]	47.3±29.5[b]
IL-12(p70)	2.7±0.3[a]	15.1±11.3[a]	196.9±85.5[b]
IL-13	3.3±0.4	3.9±0.6	28.7±13.4
IL-15	7.1±1.8[a]	8.5±3.1[a]	81.3±35.3[b]
IL-17	0.5±0.1[a]	2.1±1.1[a]	39.3±19.1[b]
CXCL-10	30.5±3.8[a]	57.9±7.4[ab]	72.1±8.8[b]
CXCL-1	28.6±11.7	25.9±2.4	42.6±7.2
MCP-1	10.5±3.4[a]	17.0±6.9[a]	95.7±37.2[b]
MIP-1α	31.4±5.7[a]	26.4±3.5[a]	79.5±14.5[b]
MIP-1β	18.3±2.5[a]	8.0±1.5[b]	24.8±6.5[ab]
MIP-2	28.7±6.4[a]	60.0±15.3[a]	157.1±36.8[b]
RANTES	1.2±0.1[a]	2.5±0.8[b]	11.1±6.8[c]
TNFα	6.3±0.5[a]	8.2±1.9[a]	30.3±7.5[b]

Data are means±s.e.m. Corresponding log-transformed data Heatmap is shown in Figure 5b. Data were analyzed by the Kruskal-Wallis procedure and the Mann-Whitney U test when appropriate. Any value below the lower limit of detection of the assay was replaced by the minimal detectable concentration (MOD)/√2 for the specific analyte, and these values were ranked as a tie for purposes of the statistical analysis. Different letters denote a significant difference between groups, $P \leq 0.05$.

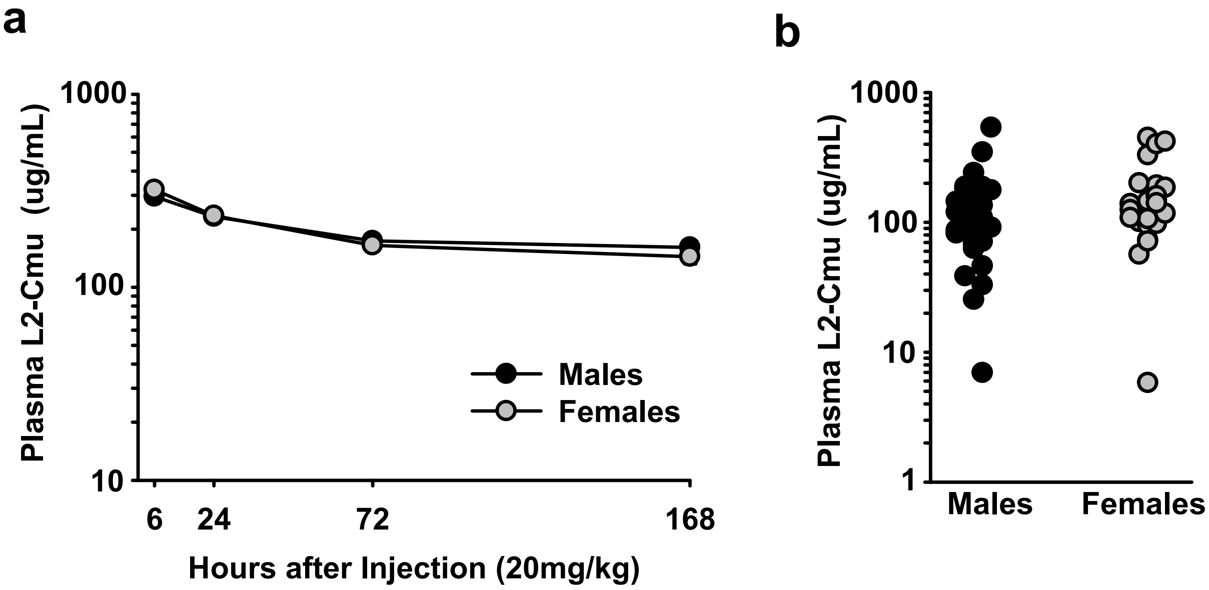

Supplementary Figure 1.

Pk and exposure levels for L2-Cmu in male and female mice.

(a) PK studies were performed in CB6F1 mice following a single i.p. injection of L2-Cmu (20mg/kg) (*n*=4-5 per group), and concentrations are presented in log scale. While L2-Cmu was undetectable in baseline control mice, L2-Cmu levels rapidly approached 300µg/mL within 6 hrs in both males and females, and levels remained >160 µg/mL at 72hrs and >140 µg/mL at 7 days, which is 5-fold greater than the reported IC_{90} values for Ganitumab (28ug/mL). Based upon these data, the calculated half-life ($t_{½}$) for L2-Cmu is 223hrs for females and 302hrs for males.

(b) For determining chronic exposure levels, plasma was obtained 48hrs following dose 24 of vehicle or L2-Cmu (20mg/kg) by i.p. injection in male Con (*n*=9) and L2-Cmu (*n*=34) and female Con (*n*=9) and L2-Cmu (*n*=30) mice, and levels are presented in log scale. While L2-Cmu levels were generally at or below the LLOQ in Controls, L2-Cmu levels were readily and similarly detectable in males (126.6±16.8µg/mL) and females (159.9±19.2µg/mL).

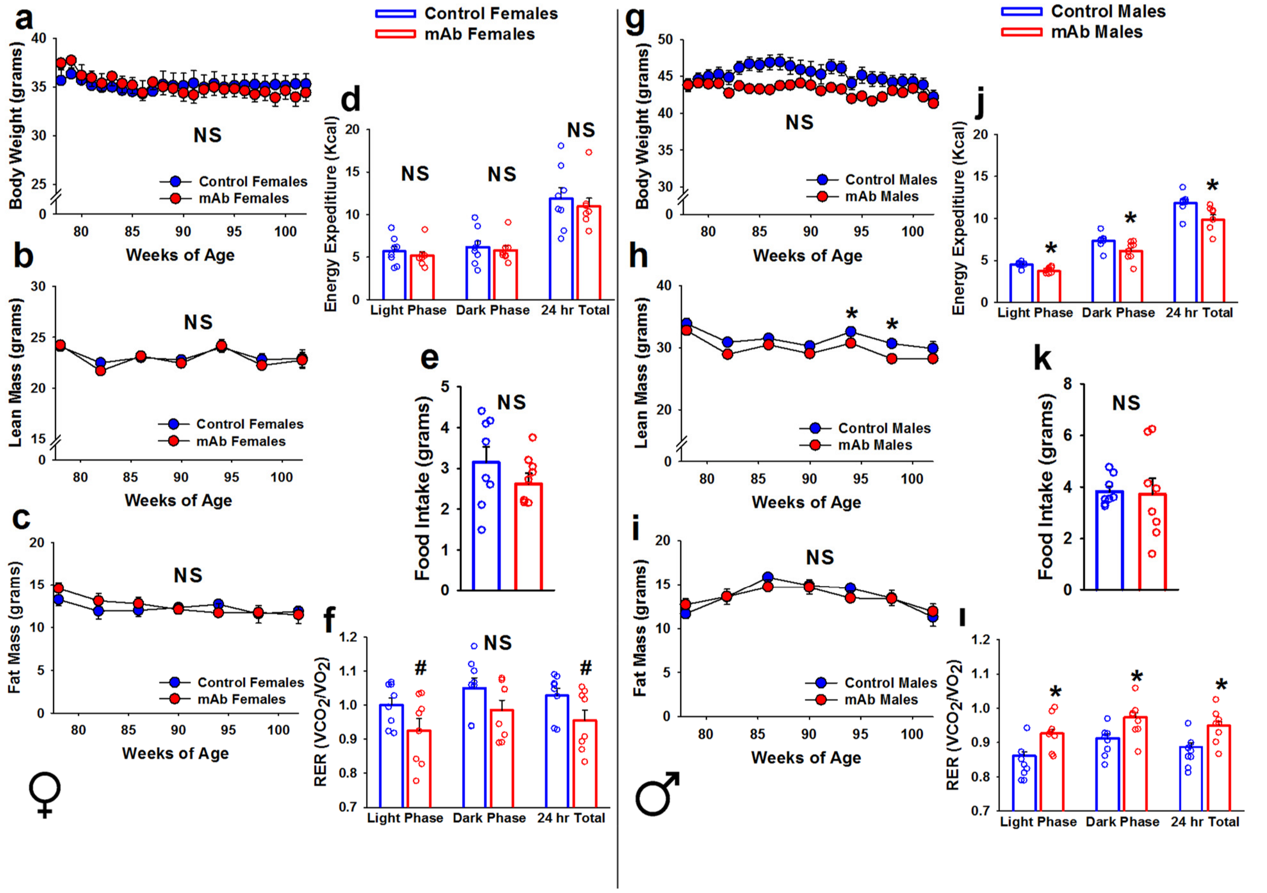

Supplementary Figure 2.

<u>Effect of L2-Cmu mAb treatment on energy balance in aged mice.</u>

(a-c) In an initial cohort of older CB6F1 female mice treated for 6 mo with vehicle or mAb, no significant effect was observed on body weight, lean mass or adiposity (*n*=23-24 per group). (**d-f**) Likewise, no effect was observed on energy expenditure, or food intake in females, although RER tended to be decreased by mAb treatment (*P*=0.07). (**g-i**) In older CB6F1 male mice, L2-Cmu tended to lower body weight and significantly reduced lean mass, without effects on adiposity (*n*=23-24 per group). (**j-l**) L2-Cmu reduced energy expenditure, without affecting food intake in males, while a significant increase in RER was observed with mAb treatment (*n*=8 per group). Lines and bars are mean±s.e.m. Dot plots overlaid on bar graphs represent individual data points. *Significantly different from Controls by independent samples t-test, *P*≤0.05; #*P*<0.07 versus Controls.

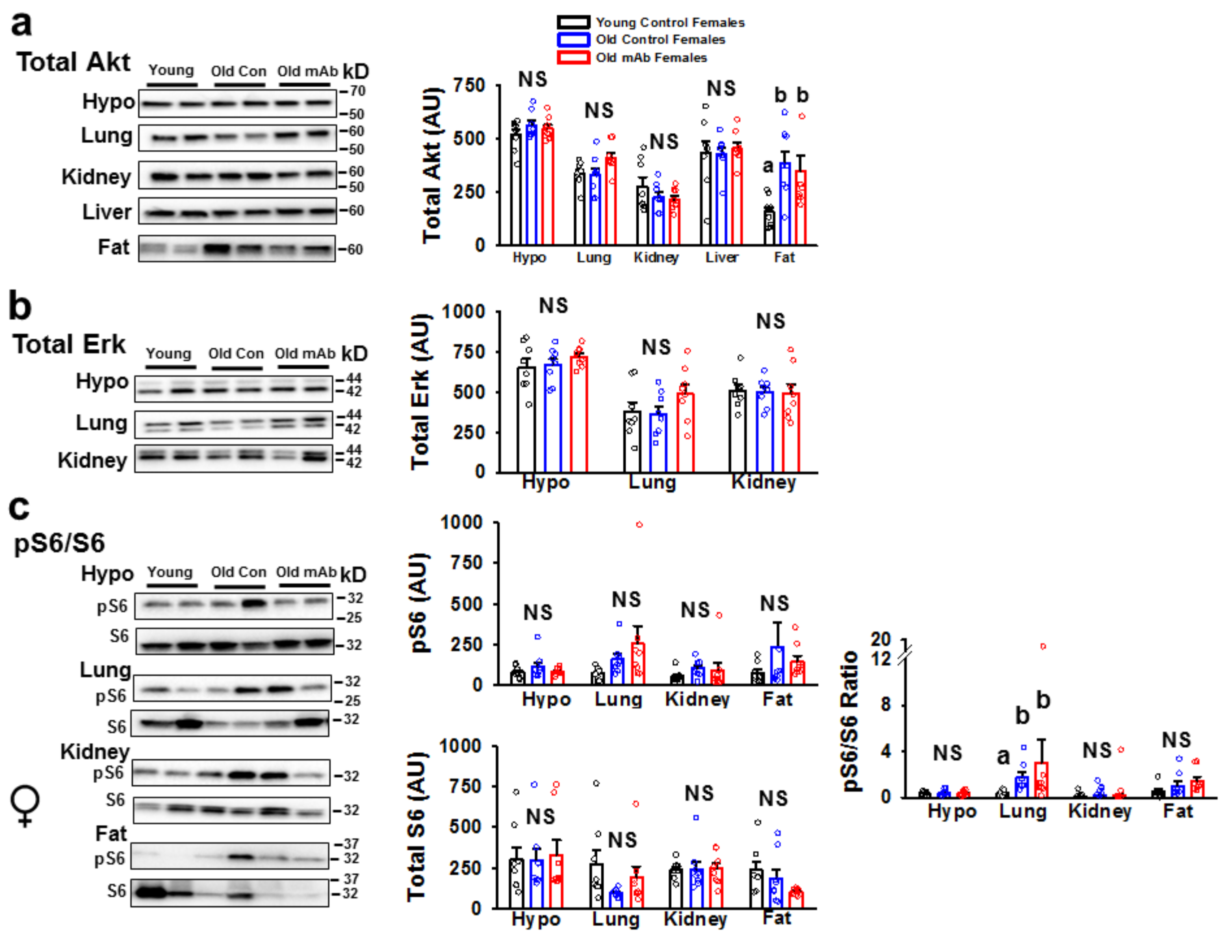

Supplementary Figure 3.

Effect of L2-Cmu mAb treatment on signaling pathway in aged female mice.

Effect of 6 mo L2-Cmu treatment on downstream components of the IGF-1 signaling pathway were evaluated in female mice at 24 mo of age (*n*=8 per group). **(a)** L2-Cmu was found to have no effect on Total Akt levels in hypothalamus (hypo), kidney, lung, liver or fat, while aging *per se* increased Akt levels in fat, regardless of treatment. **(b)** Total Erk levels were also evaluated in hypothalamus, lung or kidney, but no differences were observed in any tissue. **(c)** Activated and total S6 was also evaluated in hypothalamus, lung, kidney and fat. While no significant effect was observed on pS6 or Total S6 levels in any tissues examined, the pS6/S6 Ratio was increased with aging in lung, irrespective of treatment. Bars represent mean±s.e.m. Dot plots overlaid on bar graphs represent individual data points. NS=Not significant. Different letters denote a significant difference between groups by Tukey HSD, $P \leq 0.05$.

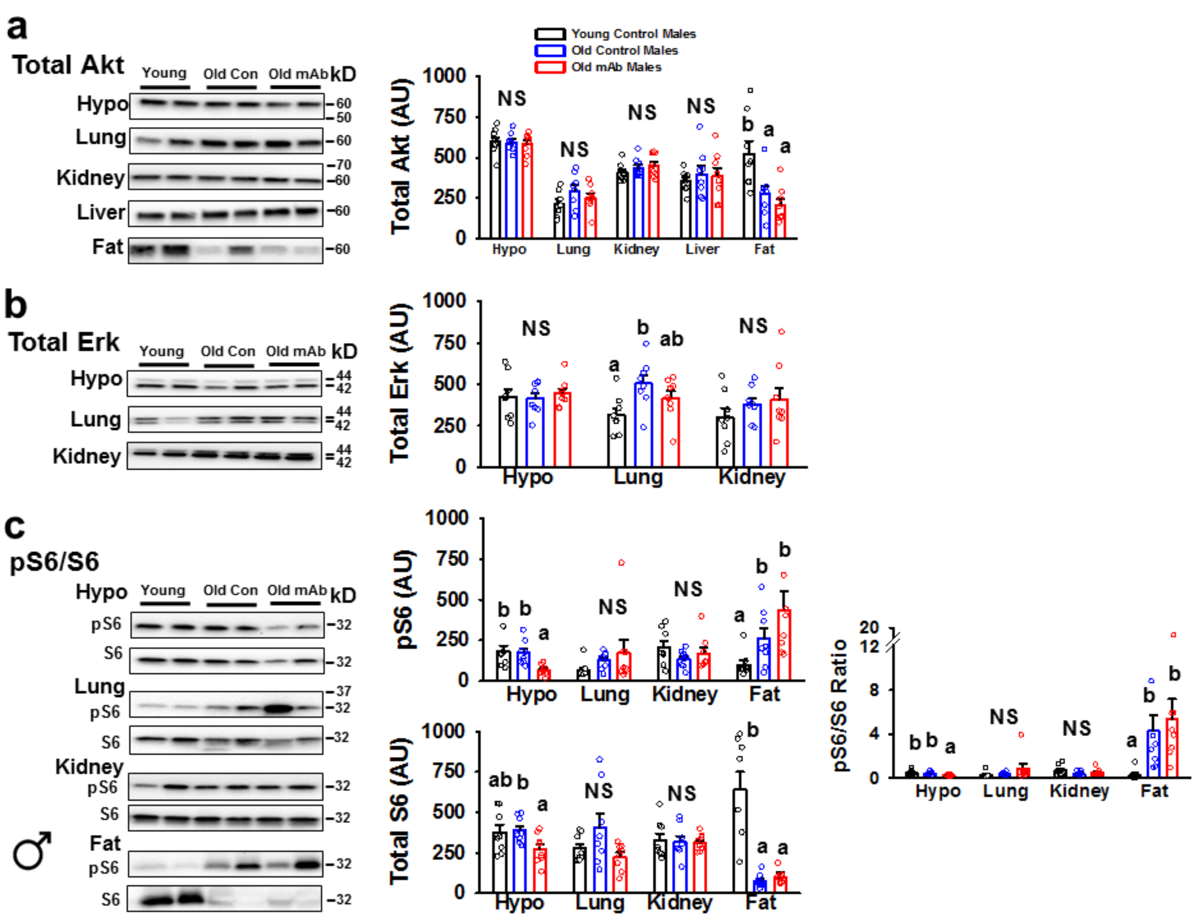

Supplementary Figure 4.

Effect of L2-Cmu mAb treatment on signaling pathway in aged male mice.

Effect of 6 mo L2-Cmu treatment on downstream components of IGF-1 signaling pathway were evaluated in male mice at 24 mo of age (*n*=8 per group). **(a)** L2-Cmu had no effect on Total Akt levels in all tissues examined, while aging *per se* led to reduced Akt levels in fat. **(b)** Total Erk levels were increased with age in lung, but L2-Cmu *per se* did not affect Erk in any tissue examined. **(c)** Activated and Total S6, as well as the pS6/S6 ratio were evaluated in several tissues, and a significant reduction was detected in pS6, Total S6, and pS6/S6 ratio for hypothalamus. Bars represent mean±s.e.m. Dot plots overlaid on bar graphs represent individual data points. NS=Not significant. Different letters denote a significant difference between groups by Tukey HSD, *P*≤0.05.

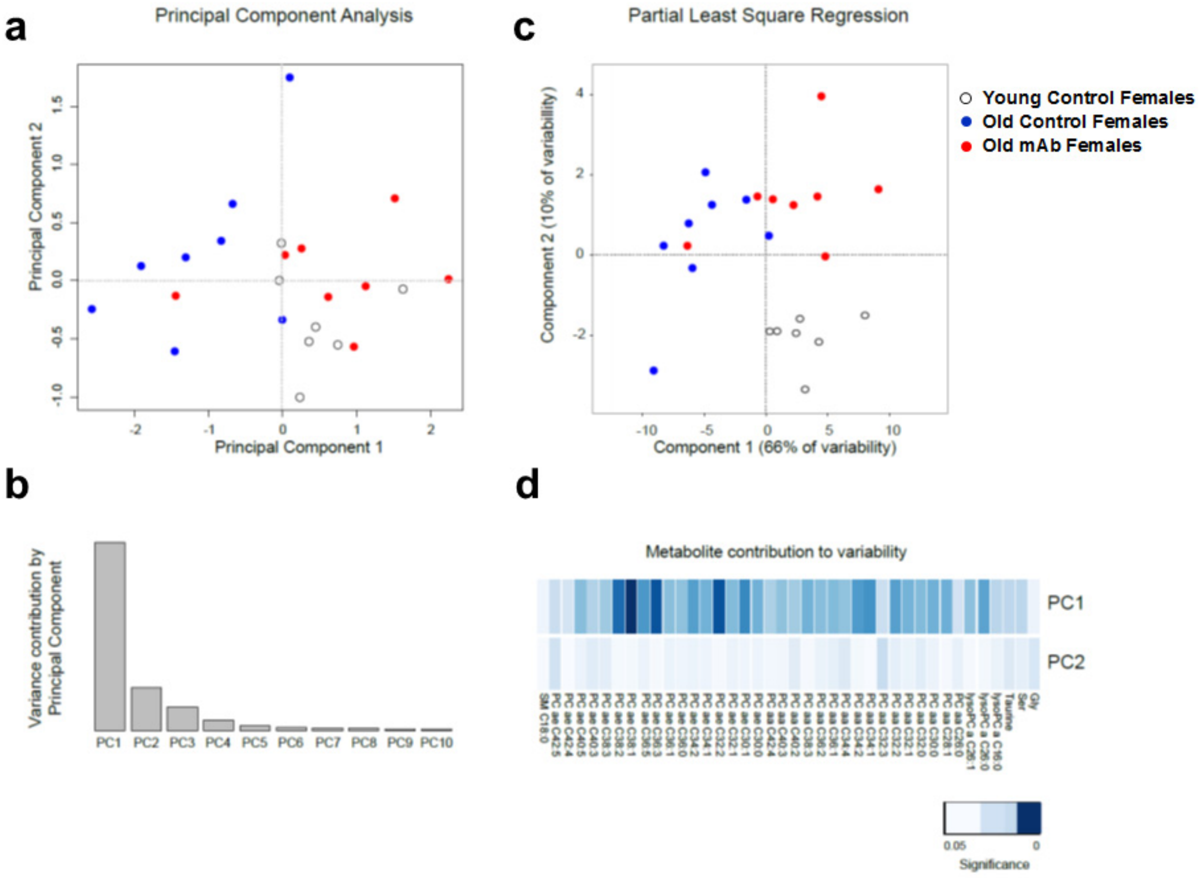

Supplementary Figure 5.

<u>PCA and PLS plots of metabolites from cardiac tissue in female mice.</u>

(**a**) PCA was performed on cardiac metabolites measured in female mice [Young (n=7), Old Con (*n*=8), and Old mAb (*n*=7)]. Based upon the generated PCA plot, cardiac metabolites from Young Con and Old mAb females tended to cluster together in the lower right quadrant, distinct from Old Con. **(b)** This effect observed by PCA was driven largely by PC1, and to a lesser extent PC2, which explained most of the variance between groups. **(c)** PLS regression plots led to a slightly different segregation of metabolites, with each group generally clustering to distinct quadrants. **(d)** Further, when linear regression was used to identify metabolite contribution to the variability between samples, PC ae C38:1 was found to be the most dissociative metabolite. Related data is presented as a heatmap is shown in Fig 4d.

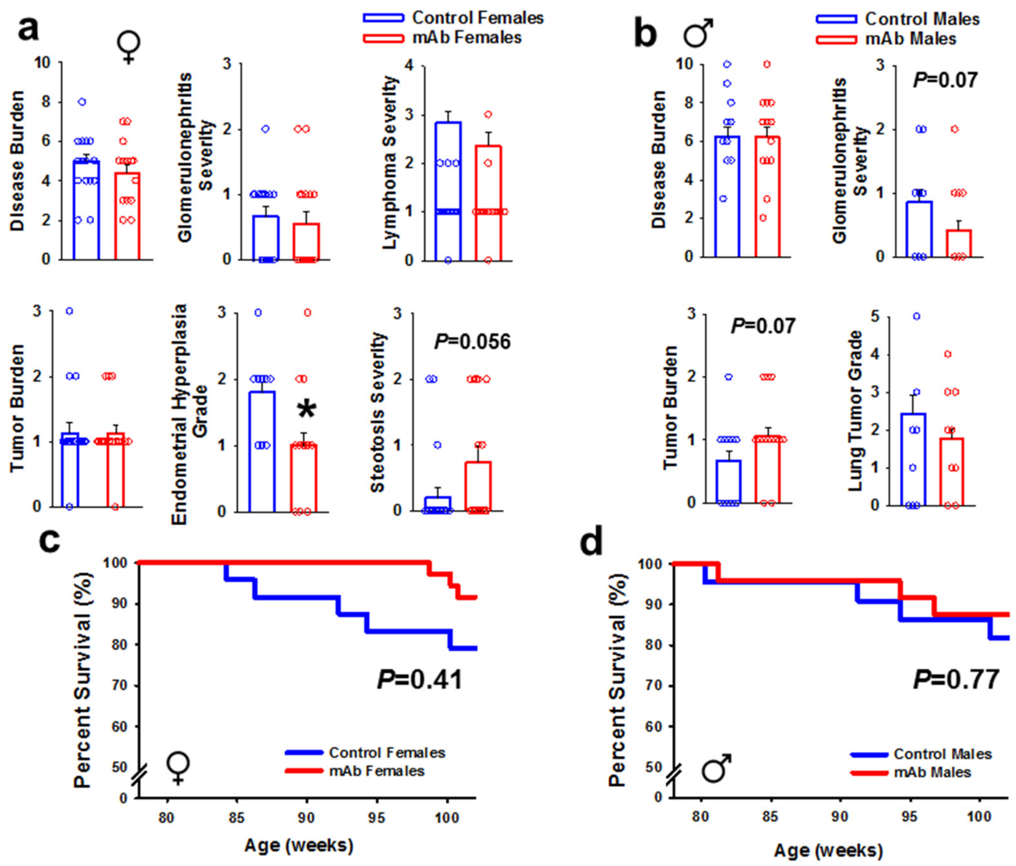

Supplementary Figure 6.

<u>L2-Cmu mAb treatment effects on interim pathology and survival.</u>

(**a-b**) Pathologic analysis was performed in 24 mo old female [Old Con (*n*=16), and Old mAb (*n*=16)] and male mice [Old Con (*n*=15), and Old mAb (*n*=17)] following 6 mo of mAb treatment. In females, mAb treatment tended to reduce endometrial hyperplasia but worsen hepatic steotosis, while treatment in males tended to reduce glomerulonephritis and increase tumor burden in males. (**c-d**) Interim survival was documented in females (*n*=24 Control and *n*=36 mAb females) and in males (*n*=36 Controls males and *n*=38 mAb males) to 24 mo old age. Female survival was suggestive of a protective effect with mAb, while male survival with L2C-mu was indistinguishable from controls. Bars represent mean±s.e.m. Dot plots overlaid on bar graphs represent individual data points. *Significantly different from Controls by chi-square test, $P \leq 0.05$.

Supplementary Figure 7.

Full Western blots from figures.

Fig. 1a

pTyr

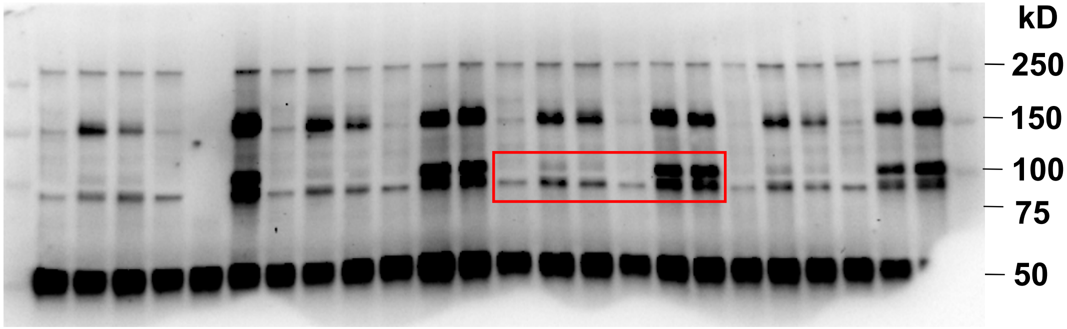

IGF-1R

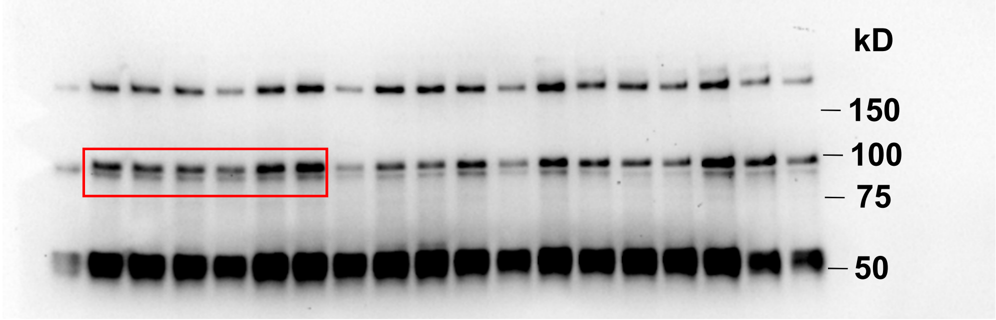

Fig. 1d

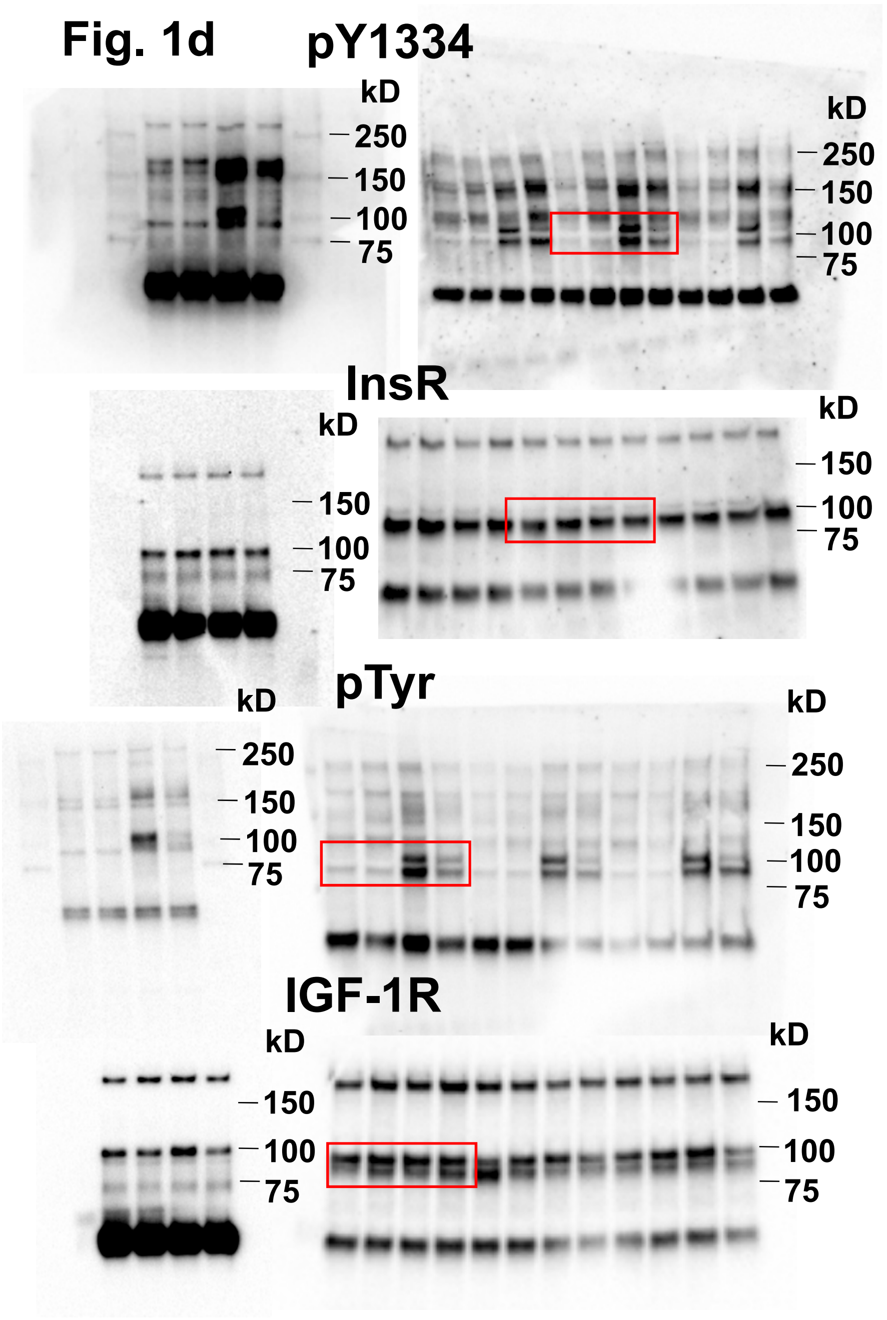
pY1334
kD
250
150
100
75
kD
250
150
100
75
InsR
kD
150
100
75
kD
150
100
75
pTyr
kD
250
150
100
75
kD
250
150
100
75
IGF-1R
kD
150
100
75
kD
150
100
75

Fig. 1e

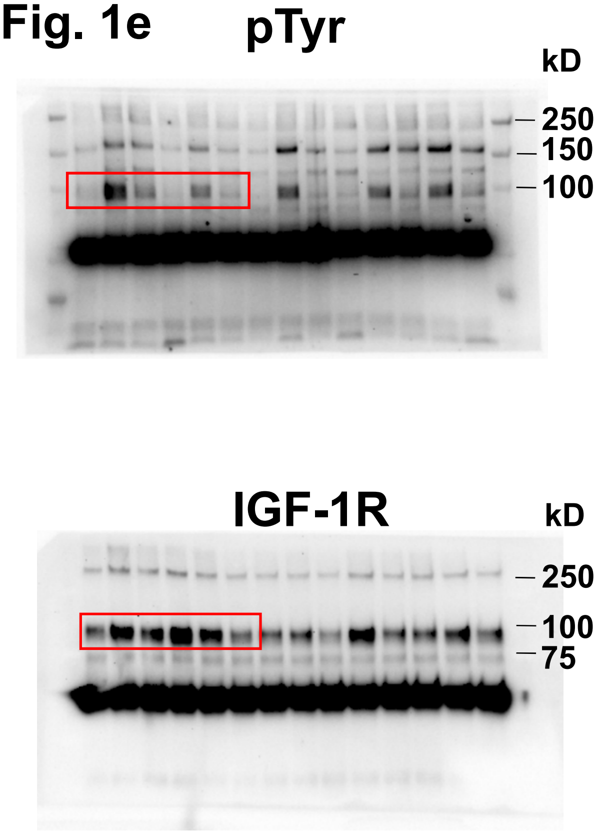

pTyr
kD
250
150
100
IGF-1R
kD
250
100
75

Fig. 1f

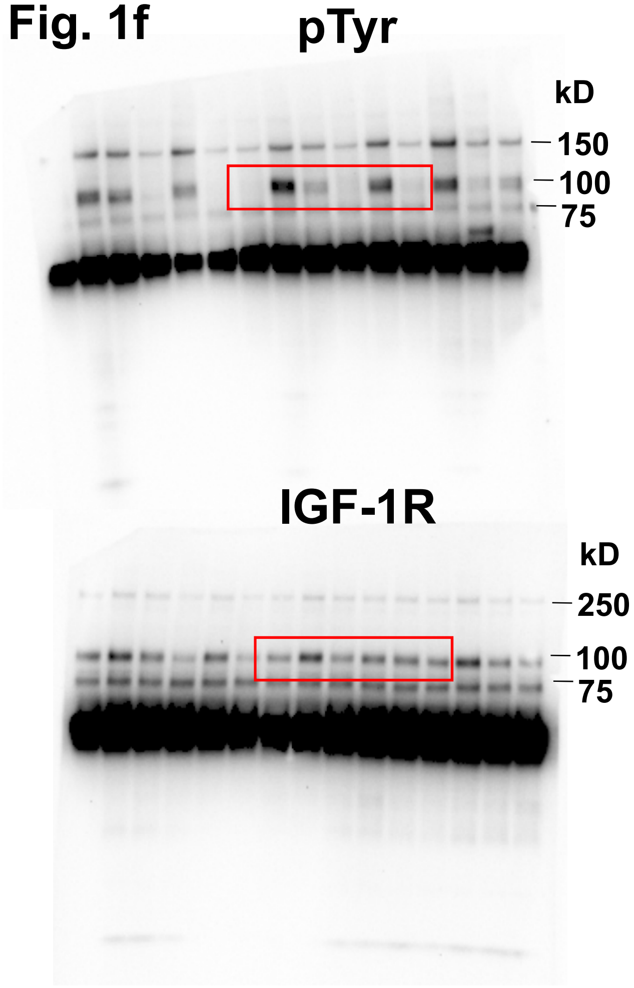

pTyr
kD
150
100
75
IGF-1R
kD
250
100
75

Fig 2i
IGF-1R♀

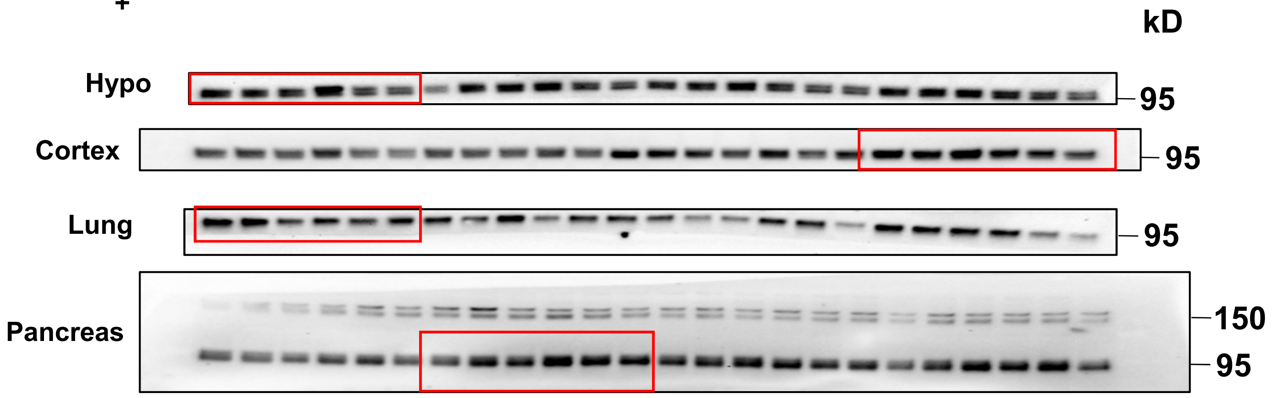

Fig 2J
IGF-1R♂

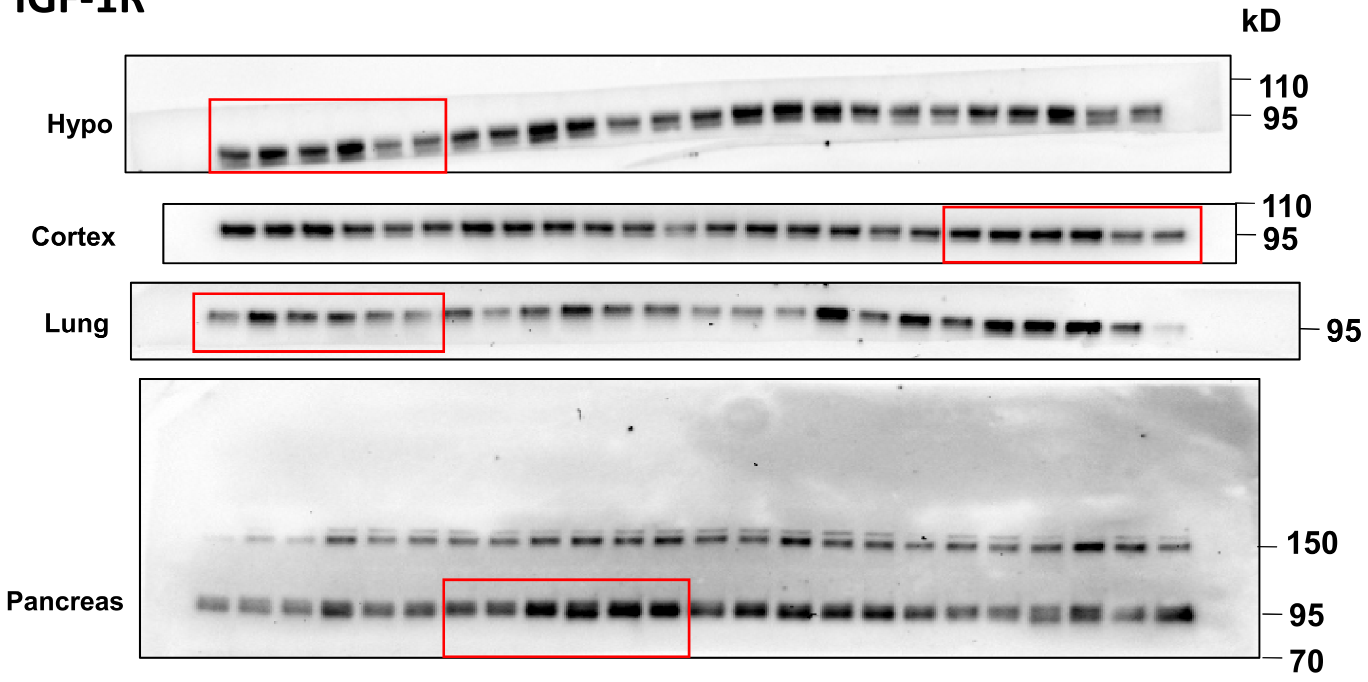

Fig 2K

InsR ♀

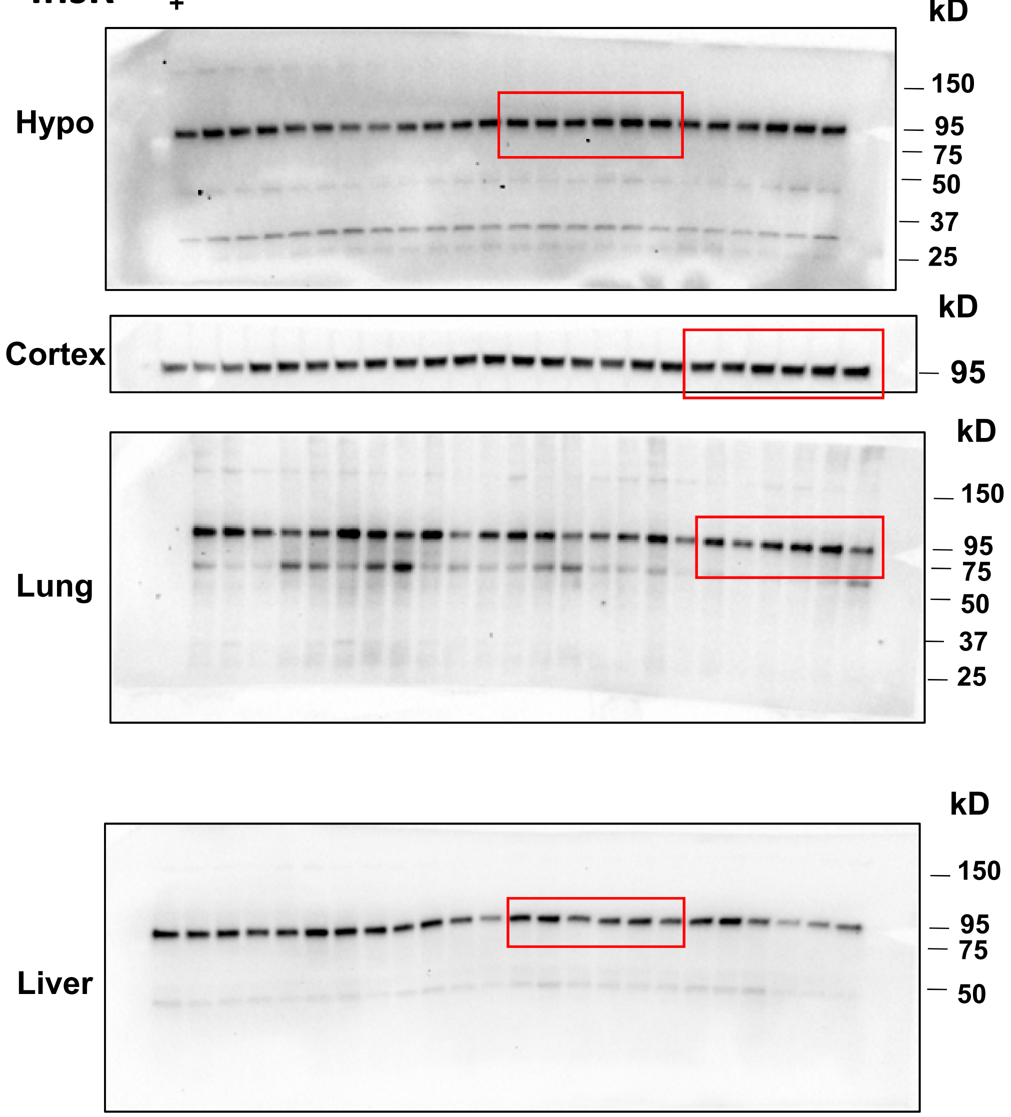

kD
Hypo
150
95
75
50
37
25
kD
Cortex
95
kD
Lung
150
95
75
50
37
25
kD
Liver
150
95
75
50

Fig 2L
InsR ♂

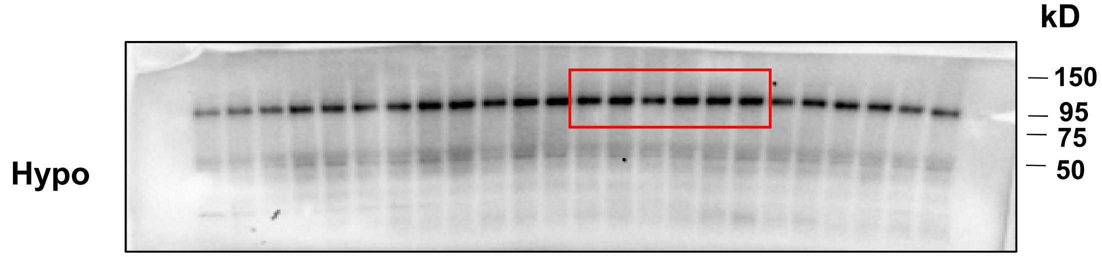
kD
150
95
75
50
Hypo

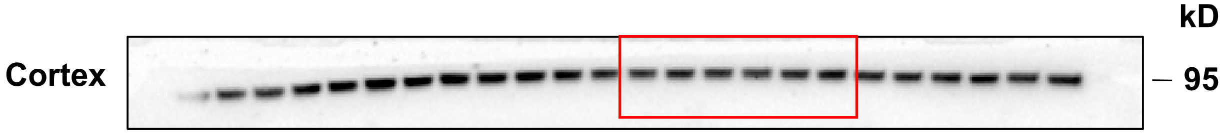
kD
95
Cortex

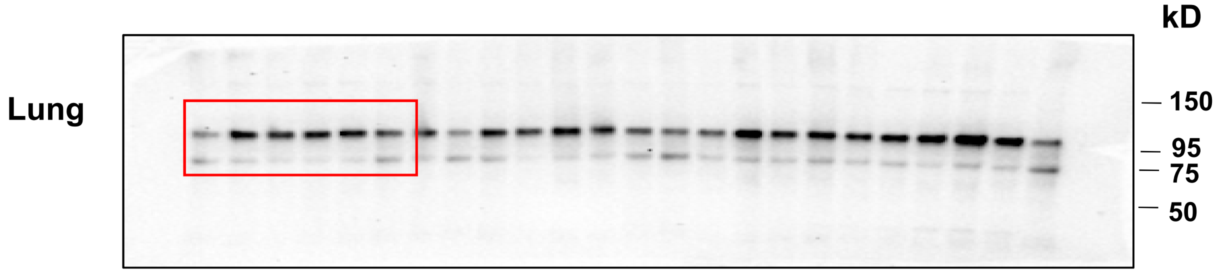
kD
150
95
75
50
Lung

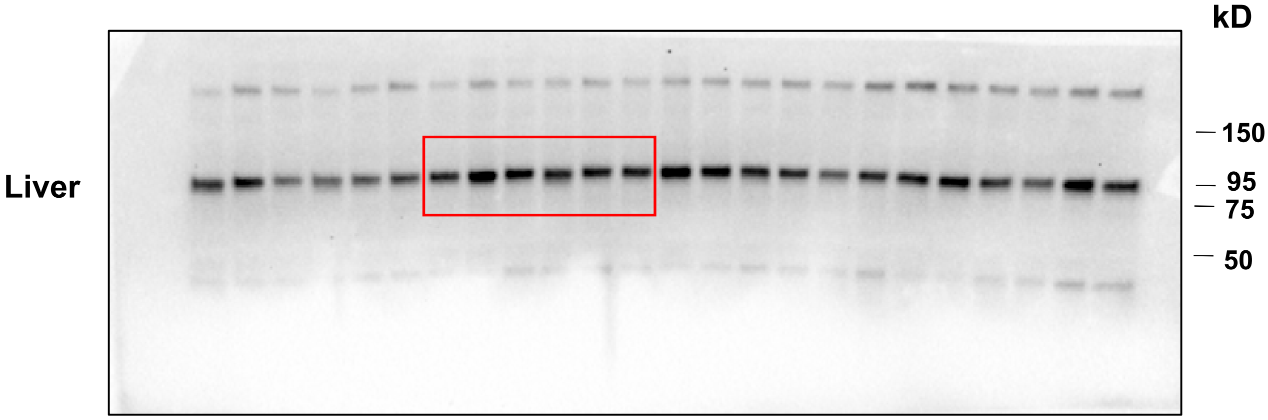
kD
150
95
75
50
Liver

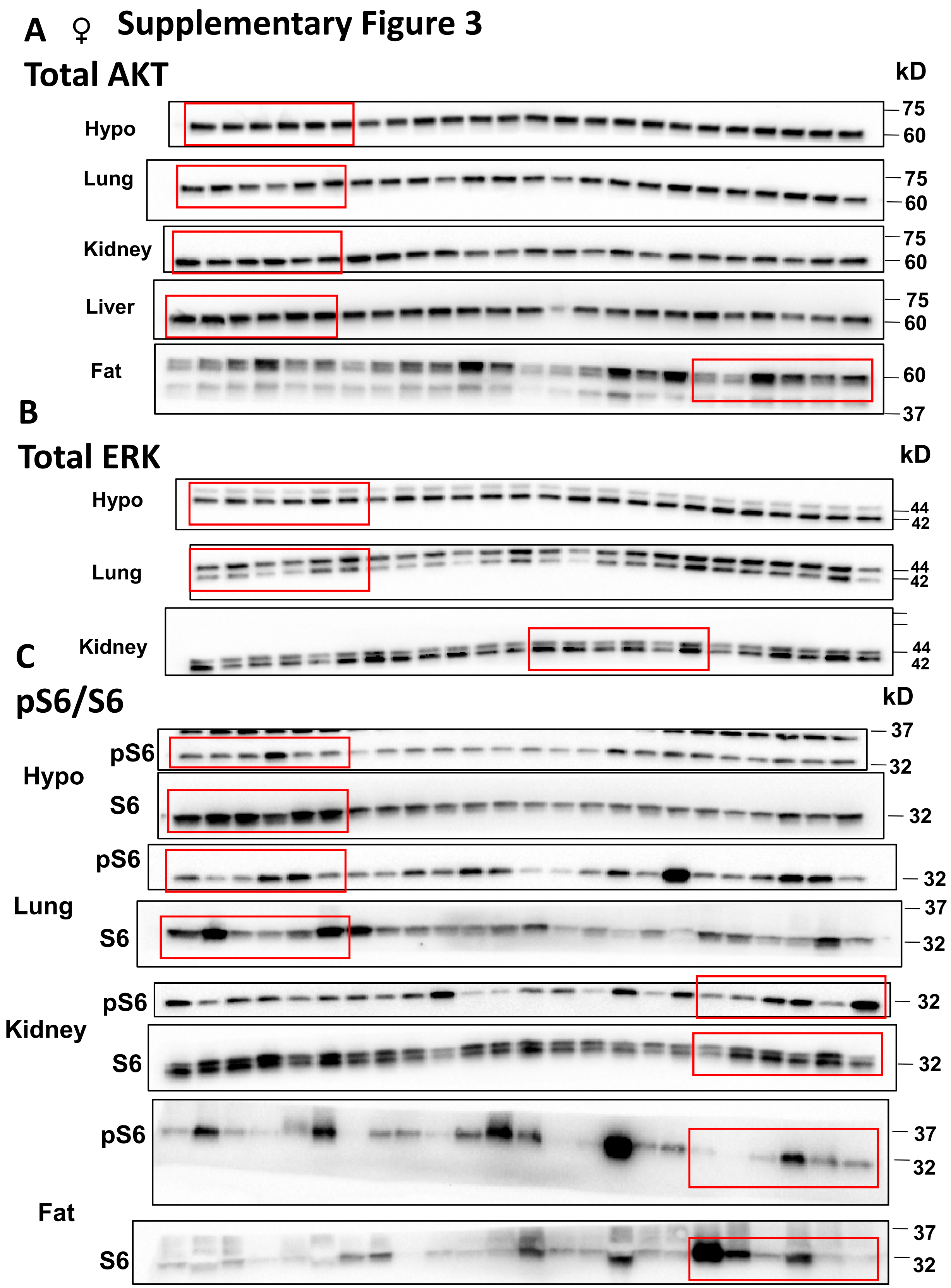

A ♀ Supplementary Figure 3
Total AKT
kD
Hypo
75
60
Lung
75
60
Kidney
75
60
Liver
75
60
Fat
60
37
B
Total ERK
kD
Hypo
44
42
Lung
44
42
Kidney
44
42
C
pS6/S6
kD
Hypo
pS6
37
32
S6
32
Lung
pS6
32
S6
37
32
Kidney
pS6
32
S6
32
Fat
pS6
37
32
S6
37
32

Supplementary Figure 4

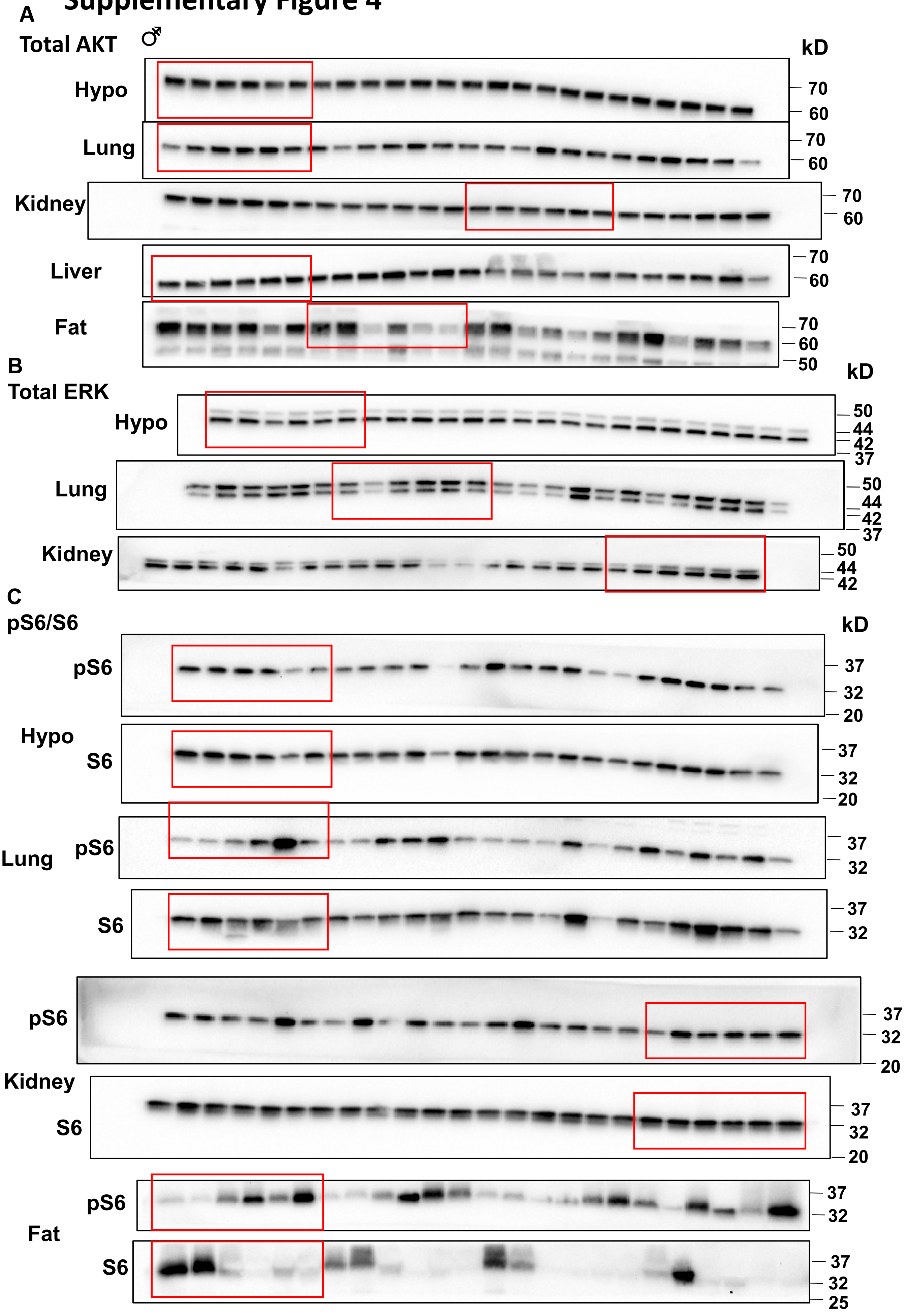

PRE-TRANSFER IMAGES

Hypothalamus ♀

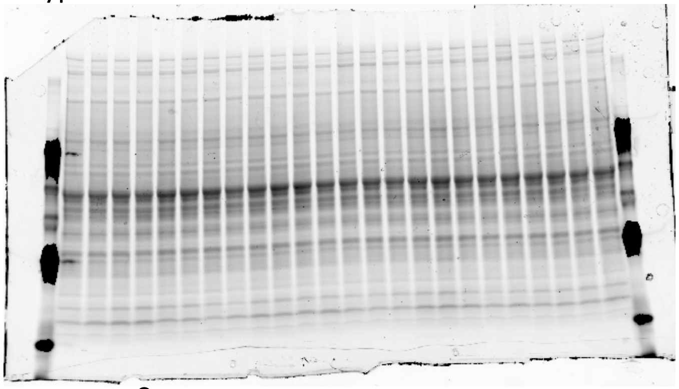

Hypothalamus ♂

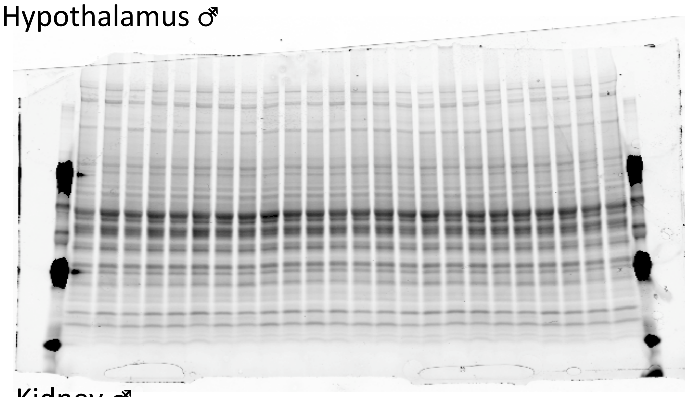

Kidney ♀

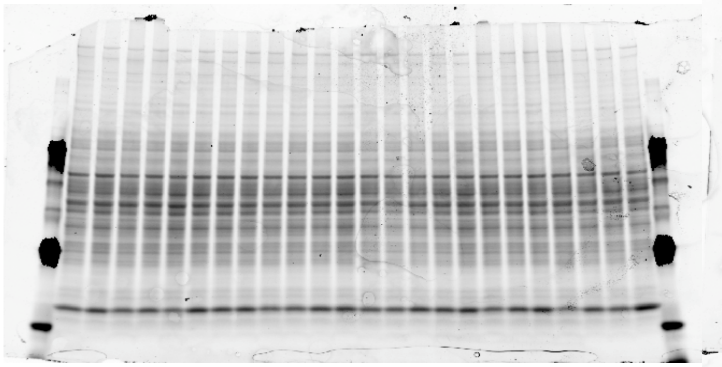

Kidney ♂

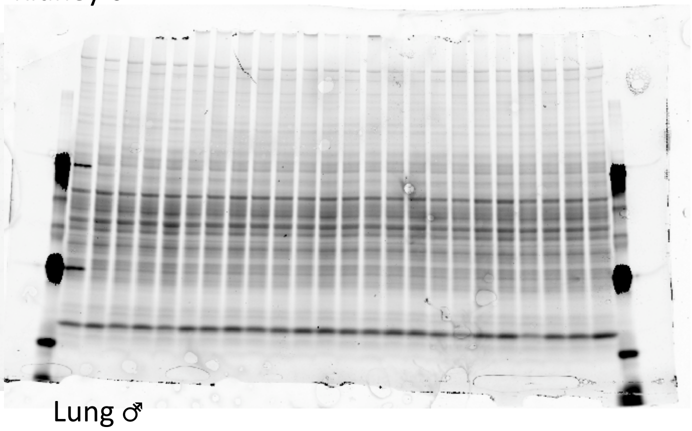

Lung ♀

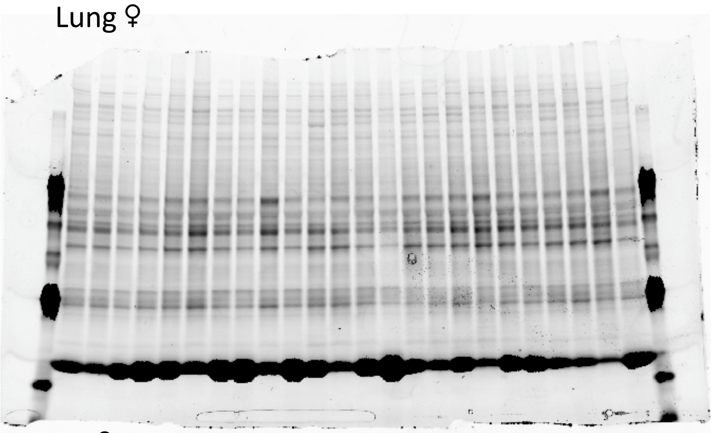

Lung ♂

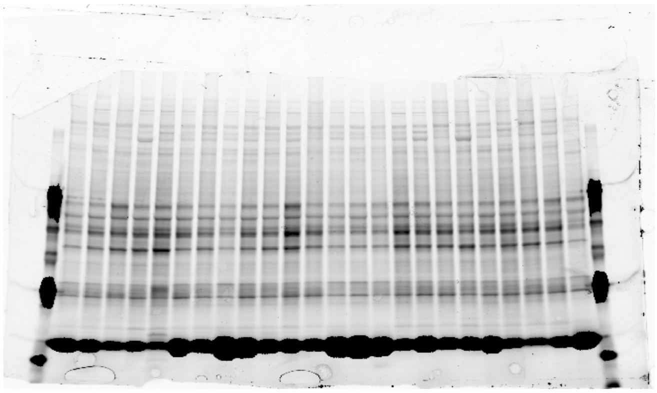

Fat ♀

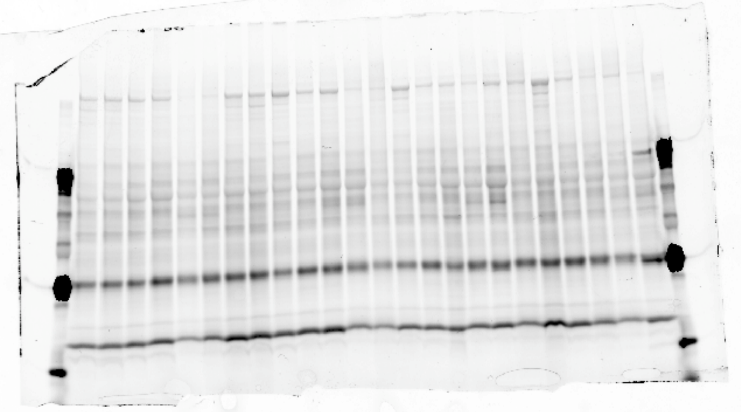

Fat ♂

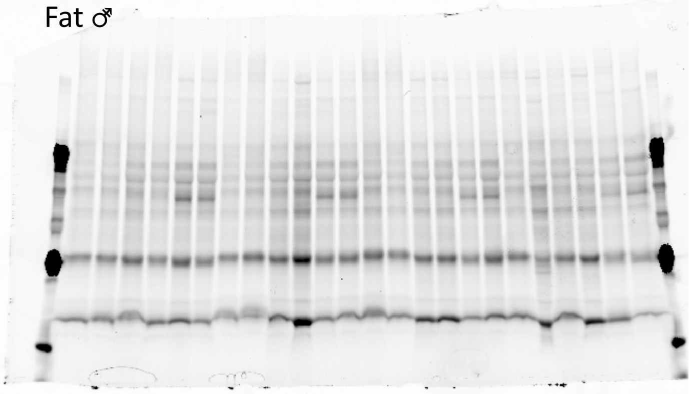

Liver ♀

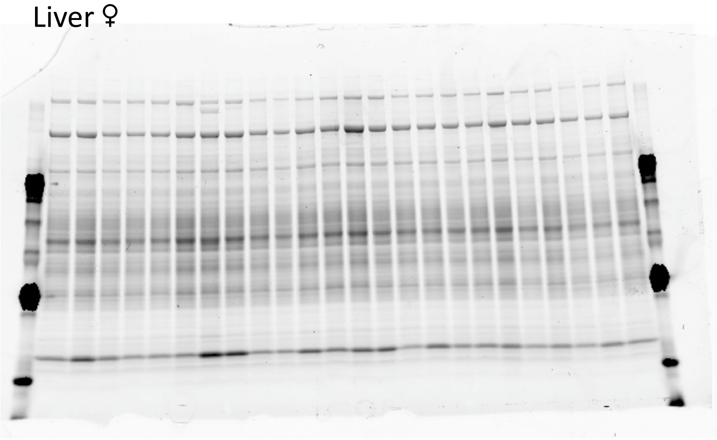

Liver ♂

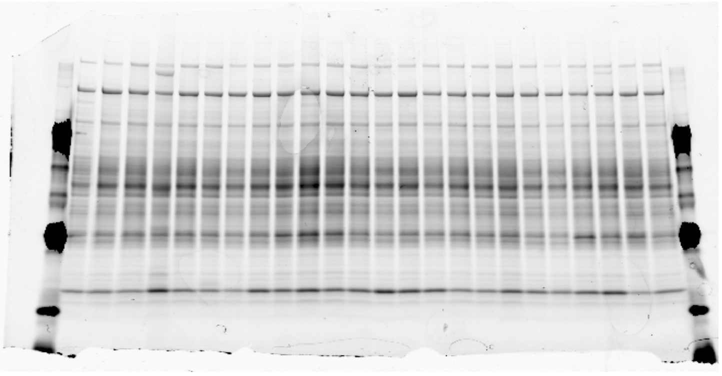

Cortex ♀

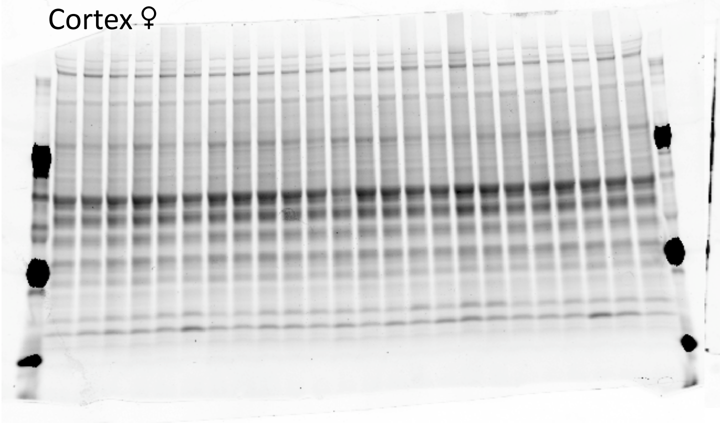

Cortex ♂

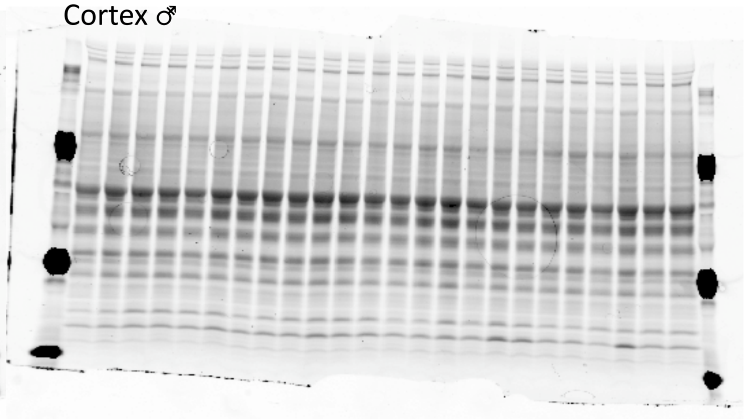

Supplement: Supplementary file 1 — Supplementary Information [file 41467_2018_4805_MOESM1_ESM.pdf]
